# A Bipartite Molecular Module Controls Cell Death Activation in the Basal Cell Lineage of Plant Embryos

**DOI:** 10.1371/journal.pbio.1001655

**Published:** 2013-09-10

**Authors:** Peng Zhao, Xue-mei Zhou, Li-yao Zhang, Wei Wang, Li-gang Ma, Li-bo Yang, Xiong-bo Peng, Peter V. Bozhkov, Meng-xiang Sun

**Affiliations:** 1 Department of Cell and Developmental Biology, College of Life Science, State Key Laboratory of Plant Hybrid Rice, Wuhan University, Wuhan, China; 2 Department of Plant Biology and Forest Genetics, Uppsala BioCenter, Swedish University of Agricultural Sciences and Linnean Center for Plant Biology, Uppsala, Sweden; University of Zurich, Switzerland

## Abstract

Plant zygote divides asymmetrically into an apical cell that develops into the embryo proper and a basal cell that generates the suspensor, a vital organ functioning as a conduit of nutrients and growth factors to the embryo proper. After the suspensor has fulfilled its function, it is removed by programmed cell death (PCD) at the late stages of embryogenesis. The molecular trigger of this PCD is unknown. Here we use tobacco (*Nicotiana tabacum*) embryogenesis as a model system to demonstrate that the mechanism triggering suspensor PCD is based on the antagonistic action of two proteins: a protease inhibitor, cystatin NtCYS, and its target, cathepsin H-like protease NtCP14. *NtCYS* is expressed in the basal cell of the proembryo, where encoded cystatin binds to and inhibits NtCP14, thereby preventing precocious onset of PCD. The anti-cell death effect of NtCYS is transcriptionally regulated and is repressed at the 32-celled embryo stage, leading to increased NtCP14 activity and initiation of PCD. Silencing of *NtCYS* or overexpression of *NtCP14* induces precocious cell death in the basal cell lineage causing embryonic arrest and seed abortion. Conversely, overexpression of *NtCYS* or silencing of *NtCP14* leads to profound delay of suspensor PCD. Our results demonstrate that NtCYS-mediated inhibition of NtCP14 protease acts as a bipartite molecular module to control initiation of PCD in the basal cell lineage of plant embryos.

## Abstract 2

During plant embryogenesis, once the suspensor organ of the plant embryo has fulfilled its role, it is removed by programmed cell death (PCD). The pro-death cathepsin protease NtCP14 initiates this PCD, but is inhibited by the cystatin NtCYS until the suspensor function is fulfilled.

## Author Summary

Similar to animals, plants eliminate tissues and organs that have transient function via programmed cell death (PCD). This is especially apparent during plant embryogenesis, when a plant zygote divides into an apical cell, which develops into a mature embryo, and a basal cell, which generates a single organ called the suspensor. During early seed development, the suspensor connects the embryo to the surrounding seed tissues and transports the nutrients and hormones required for its early development. Once its functions are fulfilled, the suspensor is subsequently eliminated by PCD. In this study, we answer a long-standing question in the field by elucidating the mechanism that is responsible for initiating suspensor PCD at a specific time during embryogenesis. Our findings show that in tobacco plant embryos, suspensor PCD is controlled by two antagonistically acting proteins—the pro-death cathepsin protease NtCP14 and its inhibitor cystatin NtCYS, which co-localize to the basal-most cell of the suspensor. High expression levels of *NtCYS* during early embryogenesis confer suspensor growth and viability by suppressing NtCP14 activity. When the suspensor ceases to grow, *NtCYS* expression is downregulated, leading to increased NtCP14 activity and to the initiation of PCD. The genetic modulation of this *NtCYS*/*NtCP14* expression ratio either delays or hastens suspensor PCD, demonstrating its indispensable role in plant embryo development.

## Introduction

Plant development begins with asymmetric division of the zygote, giving rise to two daughter cells with distinct developmental fates. A small apical cell is the founder of cell lineage generating the embryo proper, whereas a larger, basal cell establishes cell lineage leading to the embryo-suspensor. The function of the suspensor is to connect embryo to the surrounding seed tissues and to transport nutrients and growth factors to the embryo proper [1]–[5]. The suspensor is an ephemeral organ, which is not required at the advanced stages of embryogenesis and therefore eliminated, providing the earliest manifestation of programmed cell death (PCD) in plant ontogenesis [4],[5]. Dismantling of the suspensor cells is a slow process characterized by the gradual digestion of all cellular content by the growing lytic vacuoles [4], and is therefore ascribed to the class of vacuolar cell death [6]. Apart from the requirement of type II metacaspase mcII-Pa [7],[8] and short polypeptide kiss of death [9] for the execution of suspensor PCD in Norway spruce and *Arabidopsis* embryos, respectively, molecular regulation of suspensor PCD remain elusive. In particular, molecular trigger responsible for the timely initiation of cell death in the suspensor is unknown.

**Figure 1:**
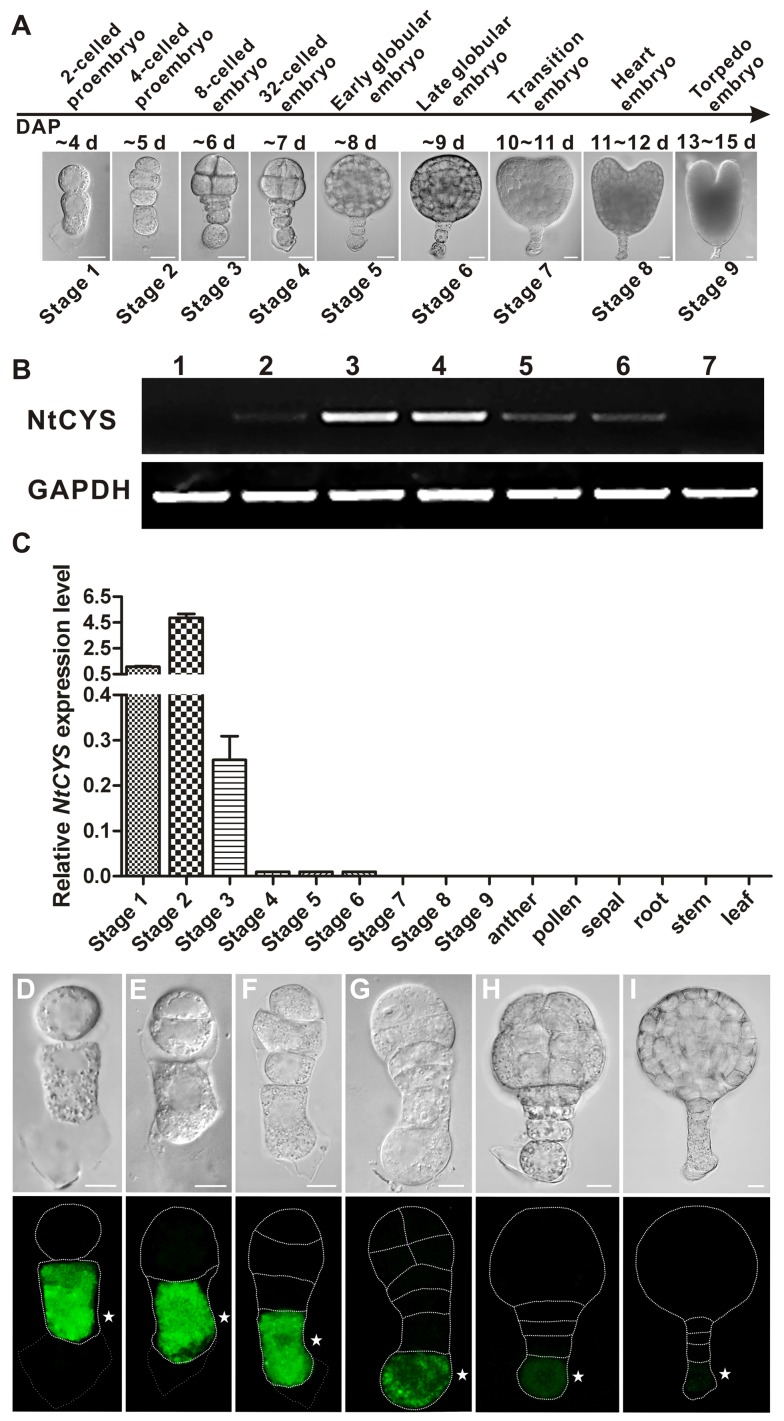
Expression pattern of *NtCYS*. (A) Classification of successive stages of embryogenesis in tobacco. DAP, days after pollination. Scale bars, 20 µm. (B) Semi-quantitative RT-PCR analysis of *NtCYS* in sperm cell (lane 1), egg cell (lane 2), zygote (lane 3), two-celled proembryo (stage 1 of embryogenesis; lane 4), eight-celled embryo (stage 3; lane 5), 32-celled embryo (stage 4; lane 6) and heart-shaped embryo (stage 8; lane 7). Glyceraldehyde-3-phosphate dehydrogenase (GAPDH) was used as a control. (C) RT-qPCR analysis of *NtCYS* in the embryos at successive developmental stages (1–9) and in both floral and vegetative tissues. The expression level of *NtCYS* in the proembryos at stage 1 was set to 1. (D–I) Localization of NtCYS-GFP at the early stages of embryogenesis in *proNtCYS*::*NtCYS-GFP* plants. (D) Two-celled proembryo (stage 1). (E) Three-celled proembryo (between stages 1 and 2). (F) Four-celled proembryo (stage 2). (G) Eight-celled embryo (stage 3). (H) 32-celled embryo (stage 4). (I) Early globular embryo (stage 5). Scale bars, 10 µm. Asterisks indicate the basal cell.

Tobacco (*N. tabacum*) embryos provide a tractable model system to address the molecular mechanisms of suspensor PCD, because of the formation of a single file of four or five suspensor cells through a highly stereotyped and precisely timed sequence of cell divisions, beginning from the first zygotic division (Figure 1A) [10]–[12]. As the first step towards the molecular understanding of the early stages of tobacco embryo development, we recently identified two groups of genes differentially expressed in the apical and basal cells of two-celled proembryos [13].

Here we demonstrate the function of one of the basal cell-specific genes, *NtCYS*, which encodes a cysteine protease inhibitor cystatin. We show that NtCYS protects the basal cell lineage from precocious initiation of cell death and is indispensable for suspensor formation and completion of embryogenesis. NtCYS exerts its anti-cell death effect by directly inhibiting cathepsin H-like protease NtCP14. Our findings establish NtCYS-dependent inhibition of NtCP14 as a bipartite molecular module regulating the fate of the basal cell lineage at the early stages of plant embryogenesis.

## Results

### NtCYS Is a New Member of the Cystatin Family in Tobacco

From a single cell transcript profile analysis designed to identify cell lineage-specific genes in two-celled proembryos of tobacco [13], we selected EST 858 (National Center for Biotechnology Information [NCBI] [http://www.ncbi.nlm.nih.gov]: HS084274, HS084275) because of its strictly basal cell-specific expression pattern. Rapid amplification of cDNA ends (RACE) followed by three rounds of genome walking produced a 683-bp cDNA and a 4,247-bp upstream region containing an ATG initiation codon. Analysis of the ORF indicated that the gene contains a complete ORF of 420 nucleotides encoding a protein of 140 amino acids. BLASTP search returned several matches to proteins containing a cystatin-like domain and a cystatin-specific QxVxG motif, indicating that the gene is a new member of the cystatin family in *N. tabacum* (Figure S1A). The gene was named *NtCYS*. The encoded protein clusters most closely with proteins from *Populus trichocarpa*, *Glycine max*, and *Medicago truncatula*, but is distant from homologs present in *Arabidopsis thaliana* and *Brassica oleracea* (Figure S1B).

Most cystatins can inhibit activity of cysteine proteases from the papain C1A family [14]. To investigate the biochemical properties of NtCYS, inhibition assays with recombinant NtCYS were carried out against papain (from papaya latex) and human liver cathepsins L and B using substrates Z-FR-AMC, Z-FR-AMC, and Z-RR-AMC, respectively. While NtCYS efficiently inhibited the activities of papain (K_i_ = 64.5±5.9 nM) and human cathepsin L (K_i_ = 0.39±0.04 nM), no measurable inhibition of human cathepsin B was detected. These data establish NtCYS as a member of the cystatin family with inhibitory activity against a subset of cysteine proteases.

### *NtCYS* Is Exclusively Expressed in the Basal Cell

To investigate the expression pattern of *NtCYS*, RNA was extracted from sperm cells, egg cells, zygotes, two-celled proembryos, and eight-celled, 32-celled, and heart embryos, and *NtCYS* expression was determined using semi-quantitative reverse transcription PCR (RT-PCR). *NtCYS* transcripts were detected in all samples, except for sperm cells and heart embryos (Figure 1B). We also assessed the levels of *NtCYS* transcripts at nine successive stages of embryogenesis, as well as in different plant organs by quantitative real-time RT-PCR (RT-qPCR). *NtCYS* was highly expressed in two- and four-celled proembryos (stages 1 and 2, respectively) and in the eight-celled embryos (stage 3), whereas the expression was barely detectable at more advanced stages of embryogenesis and was absent in non-embryonic organs (Figure 1C).

To gain insight into the spatio-temporal pattern of *NtCYS* expression, we generated transgenic plants carrying the *proNtCYS*::*NtCYS-GFP* construct. In the two-celled proembryos, NtCYS-GFP was detected only in the basal cell, but never in the apical cell (Figure 1D). When the apical cell divided, giving rise to a two-celled embryo proper, NtCYS-GFP signal continued to stay exclusively in the basal cell (Figure 1E). Surprisingly, two rounds of cell divisions in the basal cell lineage did not change the localization of NtCYS-GFP, which remained restricted to the basal cell of the two- and four-celled suspensors (Figure 1F and 1G). *NtCYS-GFP* expression markedly declined in the basal cell of the eight-celled embryos (stage 3) and was barely detectable in embryos at stage 4 and 5 (Figure 1H and 1I). These results confirm basal cell-specific expression of *NtCYS* in the two-celled proembryos and demonstrate that NtCYS-GFP has strict basipetally polarized distribution, resulting in its persistent localization in the basal cell throughout the early stages of embryogenesis.

### Suspensor PCD Starts from the Basal Cell of 32-Celled Embryos

**Figure 2:**
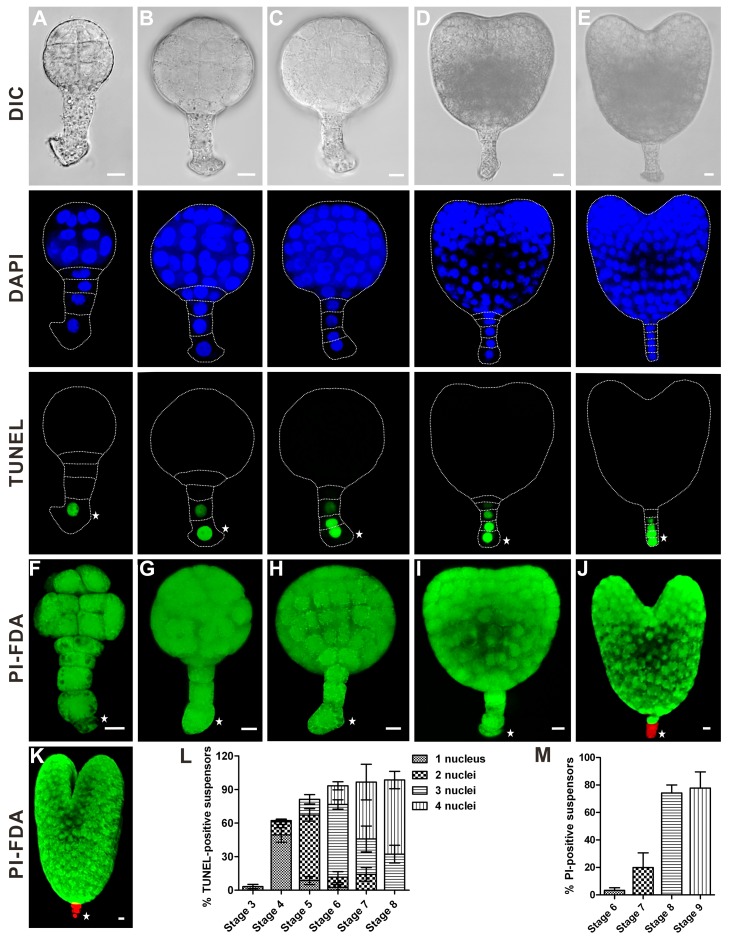
Dynamics of DNA fragmentation and plasma membrane permeabilization in the tobacco embryo-suspensor. (A–E) Nuclear DNA fragmentation in the embryos at stages 4 to 8, respectively, as revealed by TUNEL. Scale bars, 10 µm. (F–K) Plasma membrane permeabilization and cell viability of in the embryos at stages 4 to 9, respectively, revealed by FDA and PI staining. Scale bars, 10 µm. (L) The frequency of suspensors containing indicated numbers of TUNEL-positive nuclei at stages 3 to 8. Data represent the mean ± SE from five independent experiments, with 30 embryos per stage analysed in each experiment (*n* = 150). (M) The frequency of suspensors containing at least one PI-positive cell at stages 6 to 9. Data represent the mean ± SE from four independent experiments, with 30 embryos per stage analysed in each experiment (*n* = 120). Asterisks indicate the basal cell.

We investigated the dynamics of suspensor PCD during a time-course of tobacco embryo development using an array of cell death markers. Following zygotic cell division, the basal cell undergoes two successive divisions, giving rise to a four-celled suspensor at the eight-celled embryo stage (stage 3; Figure 1A). Deoxynucleotidyl transferase (TdT)-mediated dUTP nick-end labeling (TUNEL) and double staining with fluorescein diacetate (FDA) and propidium iodide (PI) showed that all cells in both apical and basal cell lineages at stages 1 to 3 maintained nuclear DNA and plasma membrane integrity (unpublished data). The first TUNEL signal was detected in the enlarged basal cell of the 32-celled embryo (stage 4) (Figure 2A). During embryo development through stages 4 to 8, TUNEL staining gradually progressed from the basal cell to the uppermost cell of the suspensor (Figure 2B–2E, and 2L), Unlike TUNEL, in the most embryos analyzed PI staining was absent in the suspensor cells until stage 8 (Figure 2F–2K, and 2M). Consequently, DNA fragmentation precedes plasma membrane permeabilization in this PCD system.

**Figure 3:**
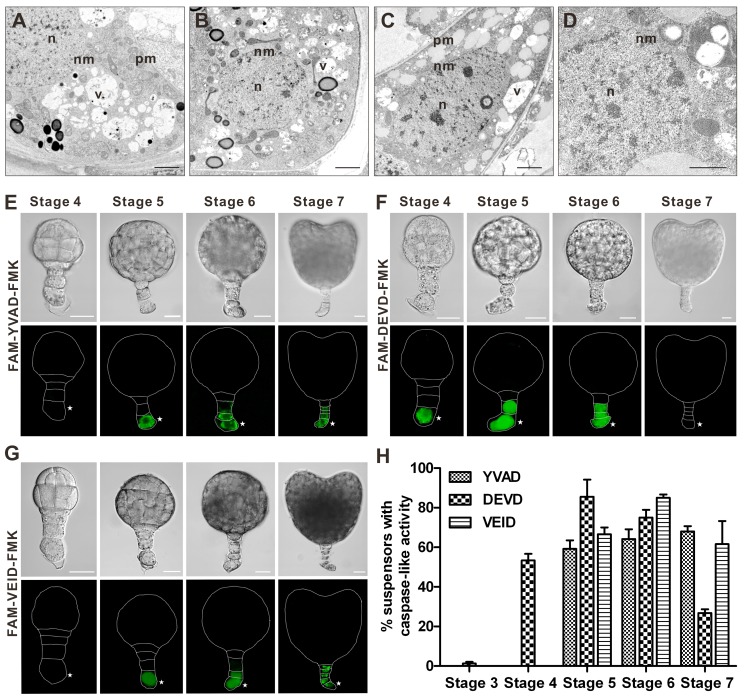
Vacuolization of the cytoplasm, nuclear envelope disassembly, and caspase-like proteolytic activity during suspensor PCD. (A–D) Morphology of the basal cell analysed by TEM in the embryos at stages 6 (A), 7 (B), 8 (C), and 9 (D). Scale bars, 2 µm in (A–C) and 1 µm in (D). n, nucleus; v, vacuole; nm, nuclear membrane; pm, plasma membrane. (E–G) *In situ* detection of active proteases with caspase 1-like (E), 3-like (F), and 6-like (G) specificity in the suspensor cells at stages 4 to 7, as revealed by staining with indicated caspase-specific fluorescent inhibitors. Scale bars, 20 µm. Asterisks denote the basal cell. (H) The frequency of suspensors with caspase-like activity at the developmental stages 3 to 7. Data represent the mean ± SE from four independent experiments, with 30 embryos per stage analysed in each experiment (*n* = 120).

We further investigated cytological features of the suspensor PCD using transmission electron microscopy (TEM). The nuclear envelope appeared intact until globular-to-heart transition (stage 7), when the nucleus became lobed and crenulated (Figure 3A and 3B). These early nuclear envelope events were followed by its progressive disassembly until complete disintegration at stage 9 (Figure 3C and 3D). Consistent with other examples of vacuolar cell death in plants [6], gradual clearance of cell contents was correlated with increased vacuolization of the cytoplasm without discernible loss of cellular turgor (Figure 3A–3D).

Recent evidence suggests the involvement of proteases with caspase 1, 3, and 6-like activities in various types of PCD in plants [15]–[20]. To establish whether caspase-like activities are involved in tobacco suspensor PCD, we studied the localization of cell-permeable fluorescent inhibitors of caspases 1, 3, and 6 in the embryos at stages 3 to 7 (Figure 3E–3H). None of these activities were detected at stage 3 (Figure 3H). The first fluorescent signal was detected at stage 4 for caspase 3-like activity, which persisted until stage 6 or 7 (Figure 3F and 3H). Caspase 1- and 6-like activities were first detected at stage 5 and remained in most of the embryos until stage 7 (Figure 3E, 3G, and 3H). Notably, the spatio-temporal pattern of all three proteolytic activities followed that of TUNEL, first appearing in the basal cell and then moving acropetally towards the uppermost suspensor cell (Figures 2A–2E and 3E–3G). No caspase-like activity was detected in the embryo proper. Together, our data demonstrate that PCD in the tobacco embryo-suspensor is a highly ordered and stereotyped process, which starts in the basal cell of 32-celled embryo (stage 4) and then progresses acropetally towards the uppermost suspensor cell adjacent to the embryo proper. This PCD exhibits cytological hallmarks of vacuolar cell death, including cytoplasm vacuolization, nuclear envelope disassembly, DNA fragmentation, and the absence of protoplast shrinkage [6], and is accompanied by the activation of proteases with caspase-like specificity.

### Downregulation of *NtCYS* Induces Precocious Cell Death in the Basal Cell Lineage and Embryo Abortion

**Figure 4:**
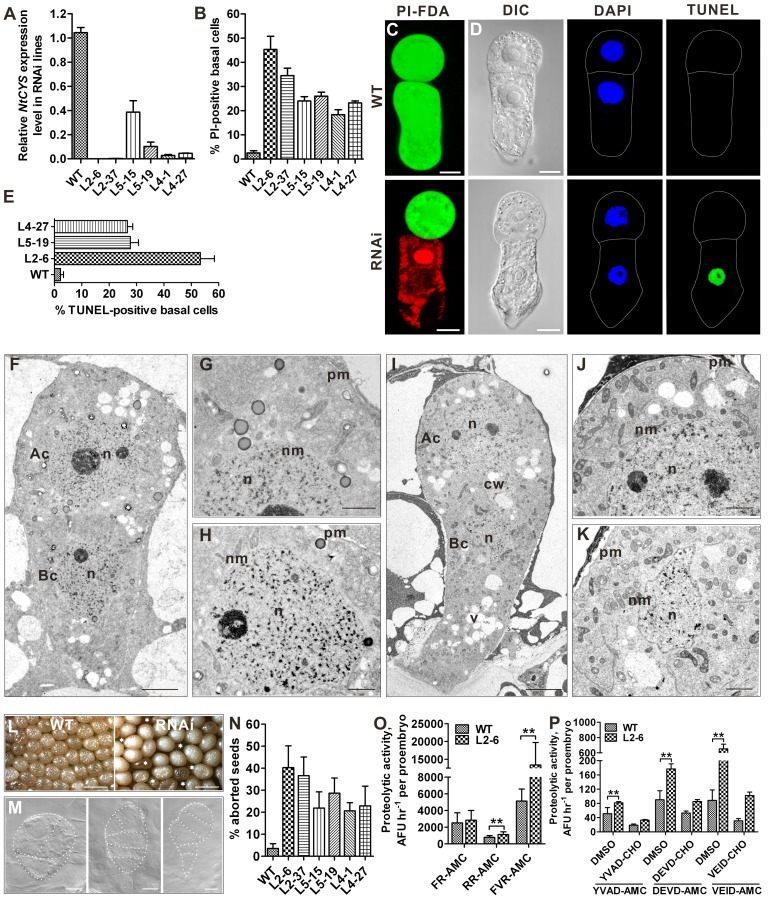
Downregulation of *NtCYS* induces precocious cell death in the basal cell lineage and seed abortion. (A) Reduced expression of *NtCYS* in RNAi lines, as measured by RT-qPCR. The expression level of *NtCYS* in the WT was set to l. (B) The frequency of the two-celled proembryos with PI-positive basal cells in WT and RNAi lines. Data represent the mean ± SE from five independent experiments, with 50 proembryos per line analysed in each experiment (*n* = 250). (C) Cell viability in the two-celled proembryos from WT and RNAi lines stained with FDA and PI. Scale bars, 10 µm. (D) Nuclear DNA fragmentation in the two-celled proembryos from WT and RNAi lines stained with TUNEL. Scale bars, 10 µm. (E) The frequency of two-celled proembryos with TUNEL-positive basal cells in WT and RNAi lines. Data represent the mean ± SE from three independent experiments, with 30 proembryos per line analysed in each experiment (*n* = 90). (F–K) Morphology of the apical (G, J) and basal (H, K) cells analysed by TEM in the two-celled proembryos from WT (F–H) and RNAi lines (I–K). Scale bars, 5 µm in (F) and (I), and 2 µm in (G, H, J and K). Ac, apical cell; Bc, basal cell; n, nucleus; v, vacuole; nm, nuclear membrane; cw, cell wall; pm, plasma membrane. (L) Seed abortion in RNAi lines. Asterisks indicate aborted seeds. Scale bars, 1 mm. (M) Aberrant cell division patterns in the apical cell lineage at the early stages of embryogenesis in RNAi lines. Scale bars, 10 µm. (N) The frequency of aborted seeds in WT and RNAi lines. Data represent the mean ± SE from three independent experiments, with 400 to 500 seeds per line analysed in each experiment. (O, P) Cathepsin-like (O) and caspase-like (P) activities in the two-celled proembryos from WT and *NtCYS* RNAi line L2-6. Data represent the mean ± SE, with 40 to 60 two-celled proembryos. ** indicates statistical difference compared to WT (*t*-test, *p*<0.01).

Since the onset of the suspensor PCD occurred concurrently with a steady decrease in *NtCYS* expression in the embryos (Figures 1 and 2), we speculated that NtCYS could control this PCD. To address this possibility we silenced *NtCYS* using three independent RNA interference (RNAi) constructs expressed under the native promoter and selected six homozygous RNAi lines for detailed analysis (Figure 4A). We observed that in contrast to wild type (WT), a substantial fraction of two-celled proembryos in these lines (18.4%–45.3% for different lines) had PI-positive and FDA-negative basal cells, indicating that they were already dead (Figure 4B and 4C). However, the apical cells in these proembryos were still alive (Figure 4C).

To further investigate premature death of the basal cells triggered by NtCYS deficiency, we evaluated nuclear integrity in the two-celled proembryos in WT and three *NtCYS*-silenced lines using TUNEL and TEM. The frequency of TUNEL in the basal cells in all three lines increased dramatically compared to the WT (Figure 4D and 4E). Furthermore, TEM revealed nuclear lobing and condensation in the basal cells but not in the apical cells of the proembryos from *NtCYS*-silenced lines (Figure 4F–4K). As a substantial part of two-celled proembryos from RNAi lines developed further to four-celled proembryos (stage 2) and then to eight-celled embryos (stage 3), we assessed whether these more advanced stages were also associated with the appearance of TUNEL in the suspensor. In contrast to the WT, where most proembryos initiated DNA fragmentation in the suspensor when the embryo proper had 32 cells (stage 4) (Figure 2), *NtCYS*-silenced lines revealed frequent occurrence of TUNEL in the suspensor at stages 2 and 3 (TUNEL frequencies 28.9% and 15.6%, respectively, *n* = 90). Precocious activation of PCD in the basal domain caused abortion of 20.7% to 40% of seeds in different NtCYS-deficient lines (Figure 4L and 4N). Among the aborted seeds analyzed in the NtCYS-deficient line L2-6, 44% of embryos were arrested at the transition from stage 1 to stage 2 and 51.7% at the transition from stage 2 to stage 3 (1,283 seeds analyzed). Cessation of embryogenesis was correlated with aberrant patterns of cell division in the apical cell lineage (Figure 4M).

Collectively, these data demonstrate that NtCYS acts to suppress cell death in the basal cell lineage throughout the early stages embryogenesis until the eight-celled embryo (stage 3). Furthermore, this anti-cell death role of NtCYS is crucial for the formation of a functional suspensor and completion of embryogenesis.

### Downregulation of *NtCYS* Activates Proteases with Cathepsin- and Caspase-Like Specificities

Since *NtCYS* encodes a cystatin, which inhibits papain and human cathepsin L *in vitro*, we investigated whether cathepsin-like activity correlates with *NtCYS* expression in tobacco proembryos. For this, we compared proteolytic activities of the total protein extracts prepared from two-celled proembryos in WT and *NtCYS* RNAi plants using the fluorescent peptides Z-FR-AMC (for cathepsin L-like proteases), Z-RR-AMC (for cathepsin B-like proteases), and Bz-FVR-AMC (for cathepsin H-like proteases). Silencing of *NtCYS* led to 1.4- and 2.6-fold increase in proteolytic activity towards Z-RR-AMC and Bz-FVR-AMC, respectively, but did not affect activity towards Z-FR-AMC (Figure 4O). This suggests that the anti-cell death effect of NtCYS in the basal cell lineage may be reliant on inhibition of cathepsin B- and/or cathepsin H-like proteases.

Normal progression of PCD in the tobacco embryo-suspensor is associated with increased caspase-like activity (Figure 3). To determine whether NtCYS deficiency-induced death of the basal cells in the two-celled proembryos implicates activation of proteases with caspase-like specificity, we measured proteolytic activity in the extracts of two-celled proembryos from WT and *NtCYS* RNAi plants using caspase substrates. Silencing of *NtCYS* induced 1.6-, 2.0-, and 7.4-fold increase in the activities of proteases with caspase 1-, caspase 3-, and caspase 6-like substrate specificity, respectively (Figure 4P). The specificity of the individual activities was confirmed by competition assays using aldehyde (CHO) caspase inhibitors specific for corresponding activities (Figure 4P). Our data corroborate that the NtCYS-inhibitable cell death pathway in the basal cell lineage involves activation of proteases with caspase-like substrate specificity.

### Molecular Targets of NtCYS

**Figure 5:**
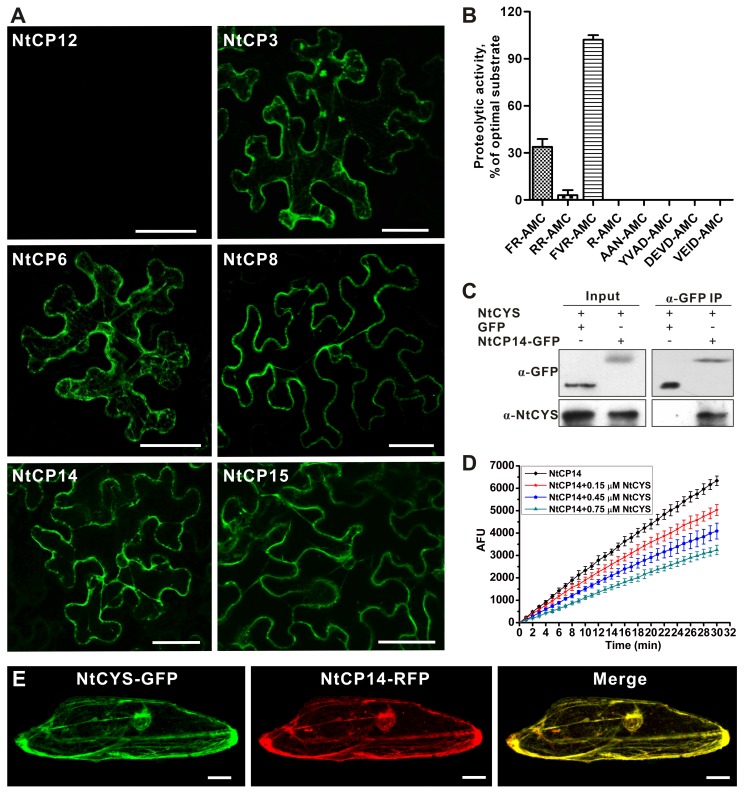
NtCYS interacts with and inhibits cathepsin H-like protease NtCP14. (A) BiFC analysis of the interaction between NtCYS and six cathepsins in tobacco epidermal cells. Scale bars, 50 µm. (B) Proteolytic activity of recombinant NtCP14 against different substrates. The activity of NtCP14 against each substrate is expressed as the percentage of NtCP14 activity against substrate FVR-AMC. Data represent the mean ± SE of three independent experiments. (C) Co-IP of NtCYS with NtCP14-GFP. GFP was used as negative control. Immunoblotting was performed with anti-NtCYS for NtCYS and anti-GFP for NtCP14-GFP or GFP. (D) NtCYS concentration-dependent inhibition of proteolytic activity of recombinant NtCP14 towards Bz-FVR-AMC. Data represent the mean ± SE of three independent experiments. (E) Cytoplasmic localization of NtCYS-GFP and NtCP14-RFP in the onion epidermal cells. Scale bars, 50 µm.

We speculated that NtCYS could directly inhibit cysteine proteases in the basal cell lineage until the 32-celled embryo stage, thereby preventing precocious activation of cell death. To retrieve cysteine protease genes from the tobacco genome, about 320,000 EST sequences were analyzed. Twenty genes encoding cysteine proteases were found to be expressed in two-celled proembryos (unpublished data). Next, we used bimolecular fluorescence complementation (BiFC) analysis in the transiently co-transfected tobacco epidermal cells to study interaction between NtCYS and the cysteine proteases, which revealed interaction with six proteases, named NtCP3, NtCP6, NtCP8, NtCP14, NtCP15 (Figure 5A), and NtCP23 (unpublished data).

Alignment of the predicted amino acid sequences showed that all six proteases contained a conserved non-contiguous ERFNIN motif (EX_3_RX_3_FX_2_NX_3_I/VX_3_N) (Figure S2), which is typical for cathepsin L- and H-like proteases, but not for cathepsin B-like proteases [21]. Next, we assessed the substrate specificities of these proteases using recombinant proteins and a panel of peptidic substrates specific for cathepsins (with Arg at the P1 position), legumains (with Asn at P1), and caspases (with Asp at P1). None of the six recombinant proteases could cleave caspase and legumain substrates, but all readily cleaved cathepsin substrates, confirming their classification as cathepsins (Figures 5B and S3).

Plant cysteine cathepsins are generally divided into four subtypes: B-, F-, H-, and L-like, which can be distinguished by their amino acid sequences and different specificities towards residues at the P2 position in their substrates [22],[23]. Considering the increased cathepsin H-like but not L-like activity found in the two-celled proembryos from the *NtCYS* RNAi plants (Figure 4O), we investigated which of the six NtCYS-interacting proteases belong to cathepsin H type by comparing their preference towards four cathepsin substrates with different P2 residues. While five out of six proteases preferred the cathepsin L substrate Z-FR-AMC (Figure S3B), a single protease, NtCP14, displayed a preference for Bz-FVR-AMC, a substrate of cathepsin H-like proteases (Figure 5B) [23]. To corroborate our BiFC results showing NtCYS interaction with NtCP14 *in vivo*, we confirmed this interaction by co-immunoprecipitation (Figure 5C). As expected, recombinant NtCYS could efficiently inhibit cathepsin H-like activity of recombinant NtCP14 (K_i(app)_ = 0.74±0.18 µM) (Figure 5D).

To gain insight into intracellular localization of NtCYS and NtCP14, we transiently expressed fluorescent proteins in onion (*Allium cepa*) epidermal cells, and found that both proteins are cytoplasmic (Figure 5E) and have strong co-localization with endoplasmic reticulum (ER) marker [24], but not with Golgi marker (Figure S4) [25]. On the basis of the above results, we chose to work further with the NtCYS-inhibitable cathepsin NtCP14, which possessed an *in vitro* P2 substrate specificity similar to that found in protein extracts from the NtCYS-deficient two-celled proembryos (Figures 4O and 5B).

The level of *NtCP14* expression was high at the early stages of embryogenesis, indicating significant overlap between *NtCP14* and *NtCYS* expression profiles (Figures 1B, 1C, S5A, and S5B). However, comparison of spatio-temporal expression patterns of two genes revealed one key difference. While *NtCYS* was expressed only in the basal cell, *NtCP14* promoter was active in both apical and basal cell lineages (Figures 1D–1I and S5C).

### Constitutive Overexpression of *NtCP14* Induces Embryonic Death at the Two-Celled Proembryo Stage

**Figure 6:**
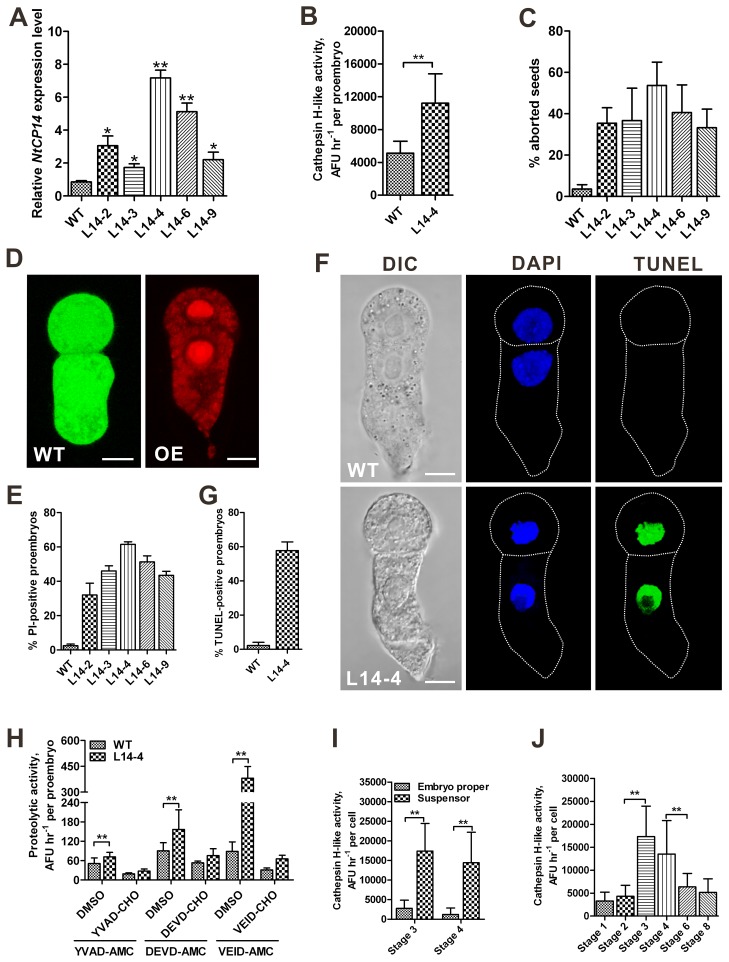
Constitutive overexpression of *NtCP14* leads to cell death at the two-celled proembryo stage. (A) Enhanced expression of *NtCP14* under the control of *proZC1* promoter in transgenic lines, as measured by RT-qPCR. The expression level of *NtCP14* in the WT was set to l. (B) Cathepsin H-like proteolytic activity towards substrate Bz-FVR-AMC in the two-celled proembryos from WT and *NtCP14*-overexpressing line L14-4. Data represent the mean ± SE, with 45 to 65 two-celled proembryos in WT and line L14-4, respectively. (C) The frequency of aborted seeds in WT and *NtCP14*-overexpressing lines. Data represent the mean ± SE from three independent experiments, with 300 to 400 seeds per line analysed in each experiment. (D) Cell viability in the two-celled proembryos from WT and *NtCP14*-overexpressing line analysed by staining with FDA and PI. Scale bars, 10 µm. (E) The frequency of two-celled proembryos with PI-positive apical and basal cells in WT and *NtCP14*-overexpressing lines. Data represent the mean ± SE from four independent experiments, with 50 proembryos per line analysed in each experiment (*n* = 200). (F) Nuclear DNA fragmentation in the two-celled proembryos from WT and *NtCP14*-overexpressing line L14-4 stained with TUNEL. Scale bars, 10 µm. (G) The frequency of two-celled proembryos with TUNEL-positive apical and basal cells in WT and *NtCP14*-overexpressing line L14-4. Data represent the mean ± SE from three independent experiments, with 30 proembryos per line analysed in each experiment (*n* = 90). (H) Caspase-like activities in the two-celled proembryos from WT and *NtCP14*-overexpressing line L14-4. Data represent the mean ± SE, with 40 to 65 two-celled proembryos in WT and L14-4, respectively. (I) Cathepsin H-like activity of NtCP14 in the WT embryo proper versus suspensor at developmental stages 3 and 4. Data represent the mean ± SE, with 85 to 100 cells of embryo proper or suspensor analysed at stages 3 and 4. (J) Cathepsin H-like activity of NtCP14 in the basal cell lineage at successive stages of embryogenesis in WT plants. Data represent the mean ± SE, with 85 to 100 suspensor cells. * and ** indicate statistical difference compared to WT or as shown otherwise (*t*-test, *p*<0.05 or 0.01, respectively).

To genetically address the role of NtCYS-inhibitable cathepsin H-like protease NtCP14 in embryogenesis, we generated tobacco lines overexpressing *NtCP14*, as well as the selected cathepsin L-like genes *NtCP6*, *NtCP8*, and *NtCP15*, under the control of the constitutive promoter *proZC1* (Figures 6A and S6). Overexpression of *NtCP14* led to a marked increase in cathepsin H-like proteolytic activity in the two-celled proembryos (Figure 6B). While embryogenesis progressed normally in the cathepsin L-like protease-overexpressing lines (unpublished data), overexpression of *NtCP14* induced massive embryonic death, resulting in the abortion of 23% to 54% of seeds in various lines (Figure 6C). Embryonic death occurred mostly at the two-celled proembryo stage, when both apical and basal cells displayed PI-positive nuclei and a lack of FDA staining (Figure 6D and 6E). Furthermore, up to 59% of the two-celled proembryos in the overexpressing lines contained TUNEL-positive nuclei in both apical and basal cells (Figure 6F and 6G). Similar to *NtCYS* RNAi plants, precocious cell death in the two-celled proembryos from *NtCP14*-overexpressing plants was accompanied by increased caspase-like activities (Figure 6H). Together, these data indicate that NtCP14 is a pro-death protease, which is able to induce cell death in both basal and apical cell lineages when overexpressed.

### NtCP14 Activation Is Restricted to the Basal Cell Lineage during the Normal Course of Embryogenesis

So far we have shown that *NtCP14* is expressed in both apical and basal cell lineages (Figure S5C) and that the increase in cathepsin H-like activity achieved through constitutive overexpression of *NtCP14* in both cell lineages induces cell death at the two-celled proembryo stage (Figure 6). A noteworthy point is that in contrast to the constitutive overexpression of *NtCP14*, its basal cell-specific overexpression using the *NtCYS* promoter did not reveal simultaneous loss of viability in apical and basal cells. Instead, we observed precocious cell death in the basal cell only, with subsequent developmental arrest and a high incidence of seed abortion (Figure S7); i.e., a phenotype similar to that of *NtCYS* RNAi lines. These data suggest that during the normal course of embryogenesis, the proteolytic activity of NtCP14 must be tightly regulated to sustain apical-basal patterning and embryo survival. To further corroborate this point, we measured cathepsin H-like proteolytic activity in the microsurgically separated WT embryo propers and suspensors at developmental stages 3 and 4 using the substrate Bz-FVR-AMC. The proteolytic activity in the suspensor was at least 6-fold of that in the embryo proper (Figure 6I). Next, we evaluated the temporal profile of cathepsin H-like activity in the basal cell lineage at successive stages of embryogenesis, beginning from the basal cell of the two-celled proembryo (stage 1) until the fully differentiated suspensor of the heart-stage embryo (stage 8). The activity in the basal cell lineage remained low in the proembryos and then increased dramatically once the embryos progressed through stages 3 (eight-celled embryo) and 4 (32-celled embryo), i.e., the stages when suspensor PCD began (Figure 6J). Importantly, the peak of the NtCP14-dependent proteolytic activity coincided with the steady decline in *NtCYS* expression level (Figure 1C), providing additional evidence for the functional role of NtCYS-mediated inhibition of NtCP14 in the regulation of the timing of suspensor PCD. Collectively, these results indicate that activation of NtCP14 in the basal cell lineage of eight- to 32-celled embryos is required for the timely onset of suspensor PCD.

### Upregulation of *NtCYS* or Downregulation of *NtCP14* Delays the Onset of Suspensor PCD

**Figure 7:**
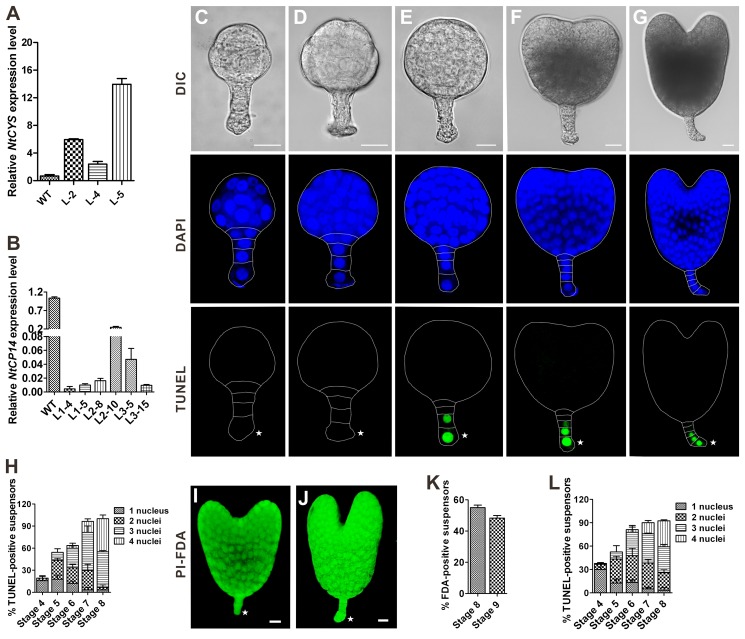
Upregulation of *NtCYS* or downregulation of *NtCP14* delays the onset of suspensor PCD. (A) Enhanced expression of *NtCYS* in overexpression lines, as measured by RT-qPCR. The expression level of *NtCYS* in the WT was set to l. (B) Decreased expression of *NtCP14* in RNAi lines, as measured by RT-qPCR. The expression level of *NtCP14* in the WT was set to l. (C–G) Representative examples of TUNEL-stained embryos in the *NtCYS*-overexpressing lines at stages 4 to 8, respectively. Scale bars, 20 µm. (H) The frequency of suspensors containing indicated numbers of TUNEL-positive nuclei in *NtCYS*-overexpressing line L-5 at stages 4 to 8. Data represent the mean ± SE from five independent experiments, with 30 embryos per stage analysed in each experiment (*n* = 150). (I,J) Plasma membrane permeabilization and cell viability in stage-8 (I) and stage-9 (J) embryos revealed by FDA and PI staining. Scale bars, 20 µm. (K) The frequency of FDA-positive suspensors at stages 8 and 9 in *NtCYS*-overexpressing line L-5. Data represent the mean ± SE from three independent experiments, with 30 embryos per stage analysed in each experiment (*n* = 90). (L) The frequency of suspensors containing indicated numbers of TUNEL-positive nuclei in *NtCP14* RNAi line L2-8 at stages 4 to 8. Data represent the mean ± SE from three independent experiments, with 30 embryos per stage analysed in each experiment (*n* = 90). Asterisks indicate the basal cell.

Because silencing of *NtCYS* or overexpression of *NtCP14* induced precocious cell death in the basal cell lineage, we argued that NtCYS-NtCP14 could act as a bipartite molecular module to control initiation of suspensor PCD. To explicitly address this hypothesis, we investigated the fate of the embryo-suspensor in *NtCYS*-overexpressing (Figure 7A) and *NtCP14* RNAi lines (Figure 7B). We found that overexpression of *NtCYS* or silencing of *NtCP14* led to a marked decrease in the level of cathepsin H-like activity in the embryos (by 35% in *NtCYS*-overexpressing line L-5 and by 30% in *NtCP14* RNAi line L2-8 compared to the WT, *n* = 150–210). In contrast to WT, a large proportion (77% to 81% for different lines) of 32-celled (stage 4) embryos in the *NtCYS*-overexpressing lines had TUNEL-negative suspensors (Figures 2L, 7C, 7H, and S8A), indicating profound delay in the onset of DNA fragmentation. Furthermore, only 42% to 45% of heart embryos (stage 8) from the *NtCYS*-overexpressing lines contained four TUNEL-positive nuclei in the suspensor (Figures 7H and S8A), as compared to 66% in the WT embryos (Figure 2L). In addition, approximately half of heart- and torpedo-stage embryos from the *NtCYS*-overexpressing lines revealed FDA-positive and PI-negative suspensors (Figure 7I–7K), which were mostly dead at the corresponding stages in the WT embryos (Figure 2J, 2K, 2M).

Similar phenotype with delayed suspensor PCD was observed in both *NtCP14* RNAi lines (Figures 7L and S8B–S8G) and lines expressing NtCYS-GFP (Figure S9). Taken together, our reverse genetic experiments demonstrate that artificial intervention in the balance between antagonistic actions of NtCYS and NtCP14 in tobacco embryos can upset (accelerate or delay) initiation of cell death in the basal cell lineage. This establishes NtCYS-NtCP14 as the bipartite molecular module that controls PCD in the embryo-suspensor.

## Discussion

Once the full-length suspensor is formed in a tobacco seed through a few rounds of transverse cell divisions, suspensor cells start dying, with the basal cell committed to death in the first place. Execution of suspensor PCD is a slow process that takes 4 to 5 d from the first signs of DNA fragmentation in the 32-celled embryo until the loss of plasma membrane integrity in the heart-stage embryo. The progression of suspensor PCD occurs in a gradient-like fashion, starting from the basal cell of the 32-celled embryo and then moving acropetally to the uppermost suspensor cell while at the same time the embryo proper undergoes rapid development (Figures 2 and 3). Notably, the formation of the PCD gradient in the embryo-suspensor seems to be an evolutionarily conserved phenomenon in plants, occurring regardless of phylogenetic position and embryo morphology, as a similar gradient of suspensor PCD has been described in a gymnosperm, Norway spruce [4],[26].

**Figure 8:**
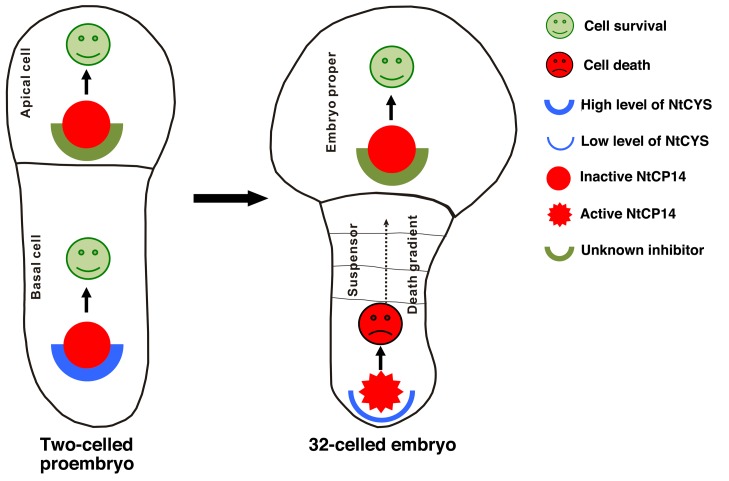
The model for the regulation of suspensor PCD by the antagonistic action of NtCYS and NtCP14.

In the present work we show that premature initiation of cell death in the basal cell lineage leads to embryo lethality. This implies that plant embryos must be equipped with a mechanism ensuring timely onset of suspensor PCD and preventing its precocious initiation. In tobacco embryos this mechanism is based on cystatin NtCYS-mediated inhibition of cathepsin H-like protease NtCP14 (Figure 8). *NtCYS* is a basal cell-specific gene asymmetrically expressed upon zygotic division (Figure 1). The expression level of *NtCYS* is high during transition from the eight-celled to the 32-celled embryo and then decreases dramatically, with simultaneous initiation of DNA fragmentation in the basal cell.

Identification of NtCP14 as an interacting protein of NtCYS explains the anti-cell death mechanism of NtCYS, which is essential for protecting the basal domain of tobacco embryos from precocious cell death. While *NtCP14* is expressed in all embryonic cells throughout the entire period of embryogenesis (Figure S5), high catalytic activity of the encoded protease is restricted to the suspensor cells of eight- and 32-celled embryos, thus correlating with the decreased *NtCYS* expression and the onset of PCD (Figures 6J and 8). We envisage NtCYS and its target protease NtCP14 as a bipartite molecular module that evolved to control PCD in the basal cell lineage (Figure 8). The balance between anti-death (NtCYS) and pro-death (NtCP14) components of the module ensures timely initiation of suspensor PCD and successful embryo development.

An intriguing question is what is the primary signal causing rapid downregulation of *NtCYS* expression and triggering suspensor PCD and where this signal comes from. Since PCD starts from the basal end of the suspensor, the signal might be derived from cells at micropyle. An alternative would be that the source of signal is the embryo proper, a scenario supported by the observations that suspensor PCD always starts when embryo proper has attained 32-celled stage. In either case, detailed investigations should be carried out to understand the nature of the signal.

Cathepsins is a collective term for a large number of structurally unrelated cysteine, serine and aspartic proteases, most of which can be found in the lysosomes or acidic vacuoles. There is a growing body of experimental evidence for the role of cathepsins B, D, and L in the execution of cell death and in the mediation of cross-talk between different types of cell death in animals [27],[28]. In contrast to animals, little is known about the role of cathepsins in plant PCD, except that cathepsin B proteases have been shown to act as pro-death components during hypersensitive response [29],[30]. Apart from regulating neurotransmitters [31] and tumor progression [32] in animals, the physiological role of cathepsin H-type proteases remained poorly understood. In the present work, we demonstrate that NtCP14 is a cathepsin H-type protease with a preference for substrate Bz-FVR-AMC and is a positive regulator of suspensor PCD. In contrast to a single cell-specific expression pattern of *NtCYS*, *NtCP14* is expressed in all embryonic cells, suggesting that the encoded protease may have other roles in embryogenesis besides regulating suspensor PCD.

While there are no close homologues of animal caspases in plants, and metacaspases (ancestral proteases found in protozoa, fungi, and plants) do not have aspartate cleavage specificity [33], at least three types of proteases with caspase-like activity exist in plants and mediate initiation and execution of PCD. These include vacuolar processing enzymes with a caspase 1-like substrate preference [16], the proteasome with caspase 3-like activity [18],[20], and a small subset of subtilisin-like serine proteases possessing caspase 6-like activity [34]. Apart from the observation that all three types of caspase-like activities correlate with the progression of suspensor PCD in WT tobacco embryos (Figure 3), we found that increase of NtCP14 activity in the two-celled proembryos achieved through genetic intervention in the NtCYS-NtCP14 module is accompanied by a corresponding increase in caspase-like activities (Figures 4 and 6). Therefore, the NtCYS-NtCP14 molecular module seems to occupy an apical position in the PCD signaling pathway, upstream to the proteases with caspase-like substrate specificity. Further studies are required to characterize and link together downstream components of the NtCYS-NtCP14 dependent PCD, in particular proteases with caspase-like specificity, metacaspases [7],[8],[33] and kiss of death peptide [9]. Unraveling of the NtCP14 degradome will also advance our understanding of the PCD pathways operating in the plant embryo-suspensor.

The very narrow, basal cell-specific expression pattern of *NtCYS* in tobacco embryos poses an intriguing question regarding how the proteolytic activity of NtCP14 is controlled in the other suspensor cells, as well as in the cells of the apical cell lineage, which are destined to survive but devoid of NtCYS (Figure 8). As suggested recently by Cambra and co-authors [35], the activities of cathepsin proteases in vivo may be controlled by several mechanisms, including local zymogene concentration and the presence of specific repertoire of cystatin inhibitors. Regardless of the actual mechanism that prevents activation of NtCP14 in the living embryonic cells, we demonstrate here that it is its local interaction with NtCYS in the basal cell that determines the life-or-death fate of both basal and apical cell lineages in the plant embryo.

## Materials and Methods

Additional details are provided in Text S1.

### Plant Materials

*N. tabacum* L. cv. Petite Havana SR1 plants were grown under 16 h/8 h light/dark cycles, at 25°C in greenhouse. *N. benthamiana* plants were grown under 14 h/10 h light/dark cycles, at 21°C in greenhouse.

### Embryo Isolation

Embryo isolation was according to our previous protocol with minor modification [11]. Detailed procedure is described in Text S1.

### Full-Length cDNA and Promoter Isolation

A full-length cDNA for *NtCYS* and cathepsin-like genes obtained using the rapid amplification of cDNA ends (RACE). Total RNA was isolated from seeds at 5 d after pollination and used as a template to synthesize first-strand cDNA with the SMART RACE cDNA Amplification Kit (Clontech). The *NtCYS* and *NtCP14* promoters were isolated using the Genome Walker DNA walking method with the Genome Walker Universal Kit (Clontech). All reactions were performed according to the manufacturer's instructions.

### Protein Sequence and Phylogenetic Analyses

A multiple sequence alignment of the known cystatin family genes in dicot plants was conducted using CLUSTALX v. 1.81 with the default multiple alignment parameters. The phylogenetic tree was constructed with PHYLIP v. 3.68.

### RT-PCR

mRNA isolation from sperm cells, egg cells, zygotes, and embryos, and cDNA synthesis were performed as described previously [13]. Total RNA was extracted from leaves, roots, stems, anthers, pollen, and sepals using TRI Reagent Solution (Ambion). All total RNAs were treated with RNase-free DNaseI (Promega), and cDNA was synthesized using Transcriptor Reverse Transcriptase (Roche) according to the manufacturer's instructions. RT-qPCR analysis was conducted as described [13] and all the data represent the mean ± standard error (SE) from three independent experiments.

### Heterologous Expression and Purification

The coding region of *NtCYS* lacking signal peptide sequence was inserted into an *Nde*I/*Xho*I -digested pMXB-10 vector (NEB). The resulting plasmid was transformed into *Escherichia coli* BL21 (DE3). The recombinant NtCYS protein was expressed and purified according to the manufacturer's instructions. The purified NtCYS was re-purified by anion exchange chromatography with a Bio-Scale Mini UNOsphere Q Cartridge (Bio-Rad) using a linear gradient of NaCl (0–300 mM) in Tris-HCl (20 mM [pH 8.0]) on BioLogic DuoFlow system (Bio-Rad). Expression and purification of cysteine proteases were performed according to [36] with minor modifications. Sequences of cysteine protease genes lacking signal peptides were inserted into the expression vector pET28a (Novagen) to yield constructs encoding proteases with both N- and C-terminal six-histidine tags. Protein expression, purification, and refolding were performed as described [36]. The final protein concentrations were quantified using a Coomassie Plus kit (Thermo) with bovine serum albumin as a standard.

### Protease Activity Assays *In Vitro* and in Cell Extracts

For determination of the K_i_ values for the interaction of NtCYS with cysteine proteases, papain (Sigma-Aldrich), human liver cathepsin L (Sigma-Aldrich), and human liver cathepsin B (Sigma-Aldrich) substrate hydrolysis progress curves were monitored. Papain activity was measured in 50 mM phosphate buffer (pH 6.0), using the synthetic fluorogenic substrate Z-FR-AMC (Sigma-Aldrich). Human cathepsin L activity was measured in 400 mM sodium acetate buffer (pH 5.5), using the synthetic fluorogenic substrate Z-FR-AMC. Cathepsin B activity was measured in 50 mM phosphate buffer (pH 6.0), using the substrate Z-RR-AMC (Sigma-Aldrich). Hydrolysis proceeded at 25°C (cathepsin L) or 37°C (papain and cathepsin B) with 40 µM substrate after or without the addition of NtCYS under reducing conditions. The activity levels were monitored using a Spectra Max M2 (Molecular Device Co.) with excitation and emission filters of 360 and 455 nm, respectively.

To determine the optimal substrates for the recombinant cysteine proteases, the proteases were activated in a buffer containing 100 mM sodium acetate (pH 4.0), and their activities were measured using substrates Z-FR-AMC (Sigma-Aldrich), Z-RR-AMC (Sigma-Aldrich), Bz-FVR-AMC (Bachem), L-R-AMC (Sigma-Aldrich), Z-AAN-AMC (Bachem), Ac-YVAD-AMC (Sigma-Aldrich), Ac-DEVD-AMC (Sigma-Aldrich), and Ac-VEID-AMC (Bachem). Hydrolysis proceeded at 30°C with 40 µM substrate in each reaction (pH 6.0) under reducing conditions. The K_i(app)_ of NtCYS for each recombinant cysteine protease was assessed according to the following equation: K_i(app)_ = [I_0_]/(V_0_/V_i_−1).

To perform endogenous protease activity assays, the embryos were frozen in 15 mM sodium phosphate or sodium acetate buffer with 0.1% Brij-35 at −80°C until use in protease assays. Proteolytic activity was measured in 10-µl reaction mixtures containing the proteins released from unfrozen embryos, and 125 µM of individual substrates, 1 mM EDTA, 10 mM L-Cys, and 0.01% Brij-35 in 15 mM sodium phosphate or sodium acetate at 30°C. The amount of AMC released was determined by capillary electrophoresis. Detailed procedure for capillary electrophoresis is described in Text S1.

### *In Situ* Caspase-Like Activity and Cell-Death Assays

Standard methods for analyzing cell viability and DNA fragmentation are also described in supplemental information Text S1.

*In situ* detection of caspase-like activity in the embryos was performed using Carboxy fluorescein FLICA Apoptosis Detection kits (Immunochemistry Technologies, LLC), which include cell-permeable fluorochrome-conjugated inhibitors of caspases. Detailed procedure is described in Text S1.

### BiFC

BiFC constructs were prepared using the vectors pSPYNE-35S and pSPYCE-35S [37]. Expression cassettes digested from pSPYNE-35S and pSPYCE-35S using *Hin*dIII and *Eco*RI were ligated into the plant transformation vector pCAMBIA1300, resulting in pCAMBIA-SPYNE and pCAMBIA-SPYCE. All full-length ORFs (without stop codons) of cysteine protease genes were inserted in-frame into the vector pCAMBIA-SPYCE. Similarly, the *NtCYS* gene was inserted into the vector pCAMBIA-SPYNE.

All BiFC constructs were transferred into *Agrobacterium tumefaciens* strain GV3101, which was used to infiltrate *N. benthamiana* leaves. The transient expression was assayed according to Sparkes [38]. Co-expression of NtCYS-SPYNE and empty vector SPYCE was used as negative control.

### Co-immunoprecipitation

NtCYS and NtCP14-GFP were co-expressed in *N. benthamiana* by *Agrobacterium*-mediated transient expression. GFP was used as the negative control. Infiltrated leaves of *N. benthamiana* were collected and ground into powder. Extracts for co-IP were prepared at 4°C in a buffer containing 15 mM Tris-HCl (pH 7.5), 150 mM NaCl, 1 mM EDTA, and protease inhibitor cocktail (1∶50; Roche). Next, a Chromotek GFP-trap (Allele Biotech) was used to capture the GFP-tagged proteins according to the manufacturer's instructions. The immunoprecipitates were then subjected to SDS-PAGE and the target protein was transferred to nitrocellulose membranes and probed with anti-NtCYS (1∶1,000) and anti-GFP (1∶2,000; Abmart). NtCYS polyclonal rabbit antiserum was produced against recombinant NtCYS (GenScript). Co-IP experiment was repeated independently three times.

### Intracellular Localization of NtCYS and NtCP14

For co-localization analysis, NtCYS-GFP and NtCP14-RFP were co-expressed in onion (*A. cepa*) epidermal cells through particle-mediated transient transformation using PDS-1000/He instrument (Bio-Rad). For subcellular localization, NtCYS-GFP and NtCP14-GFP were co-expressed with ER marker containing an N-terminal signal peptide derived from an *Arabidopsis* vacuolar basic chitinase and the C-terminal amino acid sequence HDEL (RFP-ER) [24] and Golgi marker ST-RFP (a fragment of a rat a-2,6-sialyltransferase fused to RFP; a gift from Chris Hawes, UK) [25]. Coating of gold particles and bombardment were performed according to the manufacturer's instructions (Bio-Rad Laboratories).

### Confocal Microscopy and Image Analysis

Stained embryos, transfected tobacco leaves, and transformed onion epidermis were observed under confocal microscope (Olympus FluoView FV1000). Images were processed with Image J or Adobe Photoshop.

### Transmission Electron Microscopy

For ultrastructural examination, tobacco seeds were fixed with glutaraldehyde (2.5%) in PBS buffer (100 mM [pH 7.4]) for 24 h, dehydrated in a graded ethanol series, post-fixed with OsO_4_ (0.25%) in 30% ethanol overnight and embedded in Spurr's resin. Ultrathin sections were post-stained with uranyl acetate/lead citrate and examined with a transmission electron microscope (FEI Tecnai G^2^ 20 TWIN).

### Accession Numbers

Sequence data used in this work can be found in the GenBank (http://www.ncbi.nlm.nih.gov/Genbank) under following accession numbers: *NtCYS* (KF113570), *NtCP3* (Z99173), *NtCP6* (KF113571), *NtCP8* (KF113572), *NtCP14* (KF113573), *NtCP15* (KF113574), and *NtCP23* (AB032168).

## Supporting Information

Figure S1 **Sequence alignment and phylogenetic analysis of NtCYS protein.** (A) Alignment of NtCYS with closely-related members of cystatin family, including *G. max* GmCYS-6 (accession number ACU14962), *M. truncatula* MtCYS-1 (ABD28732.1) and MtCYS-3 (ABD32914.1), and *P. trichocarpa* PtCYS-7 (XP_002308795), PtCYS-8 (XP_002307893), and PtCYS-11 (EEF07232). Identical amino acid residues are boxed. Cystatin-like domain is labelled with a shaded box. Black dots indicate QxVxG motif. (B) A bootstrap consensus of phylogenetic tree representing similarities of NtCYS protein sequence with those of *Arachis hypogaea* AhCYS (accession number: AAU21498), *A. thaliana* AtCYS-1 (AT5G12140), AtCYS-2 (AT2G31980), AtCYS-3 (AT3G12490), AtCYS-4 (AT4G16500), AtCYS-5 (AT5G47550), AtCYS-6 (At3g12490), AtCYS-7 (At5g05110), *B. campestris* BcCYS (S65071), *B. oleracea* BoCYS-1 (ABD64998), BoCYS-2 (ABD64972), BoCYS-3 (ABD64929), BoCYS-4 (AAL59842), *Castanea sativa* CsCYS (CAA11899), *G. max* GmCYS-1 (ACU14306), GmCYS-2 (CAI84599), GmCYS-3 (CAI84598), GmCYS-4 (BAA19610), GmCYS-5 (ACU19522), GmCYS-6 (ACU14962), GmCYS-7 (CAI84604), GmCYS-8 (CAI84601), *Helianthus annuus* HaCYS-1 (JE0308), HaCYS-2 (BAA95416), *Ipomoea batatas* IbCYS (AAD13812), *Malus x domestica* MdCYS-1 (AAO18638) and MdCYS-2 (AAO19652), *M. truncatula* MtCYS-1 (ABD28732.1), MtCYS-2 (ABD28593.2), MtCYS-3 (ABD32914.1), *M. sativa* MsCYS (AAZ98791.1), *Pyrus communis* PcCYS (AAB71505.1), *P. trichocarpa* PtCYS-1 (EEF09526), PtCYS-2 (EEE98761), PtCYS-3 (EEE82959), PtCYS-4 (XP_002336760), PtCYS-5 (XP_002319462), PtCYS-6 (XP_002314231), PtCYS-7 (XP_002308795), PtCYS-8 (XP_002307893), PtCYS-9 (XP_002303955), PtCYS-10 (XP_002301749), PtCYS-11 (EEF07232), PtCYS-12 (ABK94227), *Ricinus communis* RcCYS-1 (EEF36180), RcCYS-2 (XP_002525552), RcCYS-3 (XP_002523225), RcCYS-4 (EEF31214), *Solanum lycopersicum* SlCYS-1 (AAF23126), SlCYS-2 (ABG23376), SlCYS-3 (AAF23127), SlCYS-4 (ABY83981), SlCYS-5 (AAF23128), *S. tuberosum* StCYS-1 (AAA16120), StCYS-2 (ABA40456), *Vigna unguiculata* VuCYS-1 (AAQ14319), VuCYS-2 (CAA79954), *Vitis vinifera* VvCYS-1 (XP_002283400), VvCYS-2 (XP_002271940), VvCYS-3 (XP_002277752), VvCYS-4 (XP_002274494), VvCYS-5 (XP_002267841), VvCYS-6 (XP_002268378), VvCYS-7 (XP_002265736), VvCYS-8 (CAN70300), VvCYS-9 (CAN62864). The tree was calculated with Phylip Ver. 3.68. software using Protpars method. The *A. thaliana* AtCYS-6 sequence was used as an outgroup sequence in order to root the phylogenetic tree. The subclan of NtCYS is labeled with shaded box.
(TIF)

Figure S2 **Sequence alignment of tobacco cathepsins.** Identical amino acid residues are boxed. Black dots indicate non-contiguous ERFNIN motif.
(TIFF)

Figure S3 **Purification and substrate specificity of tobacco cathepsins.** (A) Coomassie blue stained SDS-PAGE gel showing purified cathepsins. (B) Proteolytic activities of recombinant cathepsins against different substrates. The activity of each cathepsin against different substrates is expressed as the percentage of its activity against substrate FR-AMC. Data represent the mean ± SE of three independent experiments.
(TIF)

Figure S4 **Co-localization analysis of NtCYS-GFP and NtCP14-GFP with Golgi and ER markers in onion epidermal cells.** While no apparent co-localization was observed with Golgi marker ST-RFP (A), both proteins showed strong co-localization with ER marker RFP-ER (B). Scale bars, 50 µm.
(TIF)

Figure S5 **Expression pattern of** ***NtCP14*** **.** (A) Semi-quantitative RT-PCR analysis of *NtCP14* in sperm cell (1), egg cell (2), zygote (3), and two-celled proembryo (4). Glyceraldehyde-3-phosphate dehydrogenase (GAPDH) was used as a control. (B) RT-qPCR analysis of *NtCP14* in the embryos at stages 1 to 9, and in both floral and vegetative tissues. The expression level of *NtCP14* in the embryos at stage 1 was set to 1. (C) Promoter activity of *NtCP14* (*proNtCP14::H2B-GFP* expression) during embryogenesis. Scale bars, 10 µm.
(TIF)

Figure S6 **Overexpression of cathepsin L-like genes driven by promoter** ***proZC1*** **.** Enhanced expression of cathepsin L-like genes in transgenic lines, as measured by RT-qPCR. The expression level in the WT was set to l, except for *NtCP8*. Data represent the mean ± SE from three independent experiments. ** indicates statistical difference compared to WT (*t*-test, *p*<0.01).
(TIF)

Figure S7 **Overexpression of** ***NtCP14*** **driven by** ***proNtCYS*** **induces precocious cell death in the basal cell lineage.** (A–C) Cell viability and nuclear DNA fragmentation at developmental stages 1 (A), 2 (B), and 3 (C) from WT and *NtCP14*-overexpressing line L1-4. Scale bars, 10 µm. (D, E) The frequency of the proembryos (stages 1 and 2) and eight-celled embryos (stage 3) with PI-positive (D) and TUNEL-positive (E) basal cells in WT and *NtCP14*-overexpressing line L1-4. Data represent the mean ± SE from three independent experiments, with 30 proembryos or embryos per line analyzed in each experiment (*n* = 90). ** indicates statistical difference compared to WT (*t*-test, *p*<0.01).
(TIF)

Figure S8 **Upregulation of** ***NtCYS*** **or downregulation of** ***NtCP14*** **delays the onset of suspensor PCD.** (A) The frequency of suspensors containing indicated numbers of TUNEL-positive nuclei in *NtCYS*-overexpressing line L-2 at stages 4 to 8. Data represent the mean ± SE from three independent experiments, with 30 embryos per stage analyzed in each experiment (*n* = 90). (B) The frequency of suspensors containing indicated numbers of TUNEL-positive nuclei in *NtCP14* RNAi line L3-15 at stages 4 to 8. Data represent the mean ± SE from three independent experiments, with 30 embryos per stage analyzed in each experiment (*n* = 90). (C–G) Representative examples of TUNEL-stained embryos in the *NtCP14* RNAi lines at stages 4 to 8, respectively. Scale bars, 20 µm.
(TIF)

Figure S9 ***NtCYS-GFP*** **expression delays the onset of suspensor PCD.** (A) Expression of *NtCYS-GFP* driven by *proZC1* in two-celled proembryos. Scale bars, 10 µm. (B) The frequency of suspensors containing indicated numbers of TUNEL-positive nuclei in *NtCYS-GFP* expressing line L-1 at stages 4 to 6. Data represent the mean ± SE from three independent experiments, with 30 embryos per stage analyzed in each experiment (*n* = 90). (C) The frequency of suspensors containing indicated numbers of TUNEL-positive nuclei in *NtCYS-GFP* expressing line L-2 at stages 4 to 6. Data represent the mean ± SE from three independent experiments, with 30 embryos per stage analyzed in each experiment (*n* = 90). (D–F) Representative examples of TUNEL-stained embryos in the *NtCYS-GFP* expressing lines at stages 4, 5, and 6, respectively. Scale bars, 10 µm. Asterisks indicate the basal cell.
(TIF)

Figure S10 **Expression of** ***NtCP14-GFP*** **induces precocious cell death.** (A) The mRNA level of *NtCP14* in *pNtCP14::NtCP14-GFP* transgenic lines. The expression level of *NtCP14* in WT was set to l. (B) The frequencies of PI-positive two-celled proembryos in WT and *NtCP14-GFP* expression lines. Data represent the mean ± SE from three independent experiments with 50 proembryos per line analyzed in each experiment (*n* = 150). (**C**) PI-FDA stained two-celled proembryos from WT and *pNtCP14::NtCP14-GFP* line. Scale bars, 10 µm.
(TIF)

Text S1 **Additional details for methods.**
(DOC)

## Abbreviations

BiFC: bimolecular fluorescence complementation
ER: endoplasmic reticulum
FDA: fluorescein diacetate
IP: immunoprecipitation
PCD: programmed cell death
PI: propidium iodide
RNAi: RNA interference
RT-qPCR: quantitative real-time reverse transcription-PCR
SE: standard error
TEM: transmission electron microscopy
TUNEL: deoxynucleotidyl transferase (TdT)-mediated dUTP nick-end labeling
WT: wild type
