# Supplementary figures and images for: A Bipartite Molecular Module Controls Cell Death Activation in the Basal Cell Lineage of Plant Embryos
Source: PLoS Biol. 2013 Sep 10;11(9):e1001655. doi: 10.1371/journal.pbio.1001655 (PMC3769231; doi:10.1371/journal.pbio.1001655)

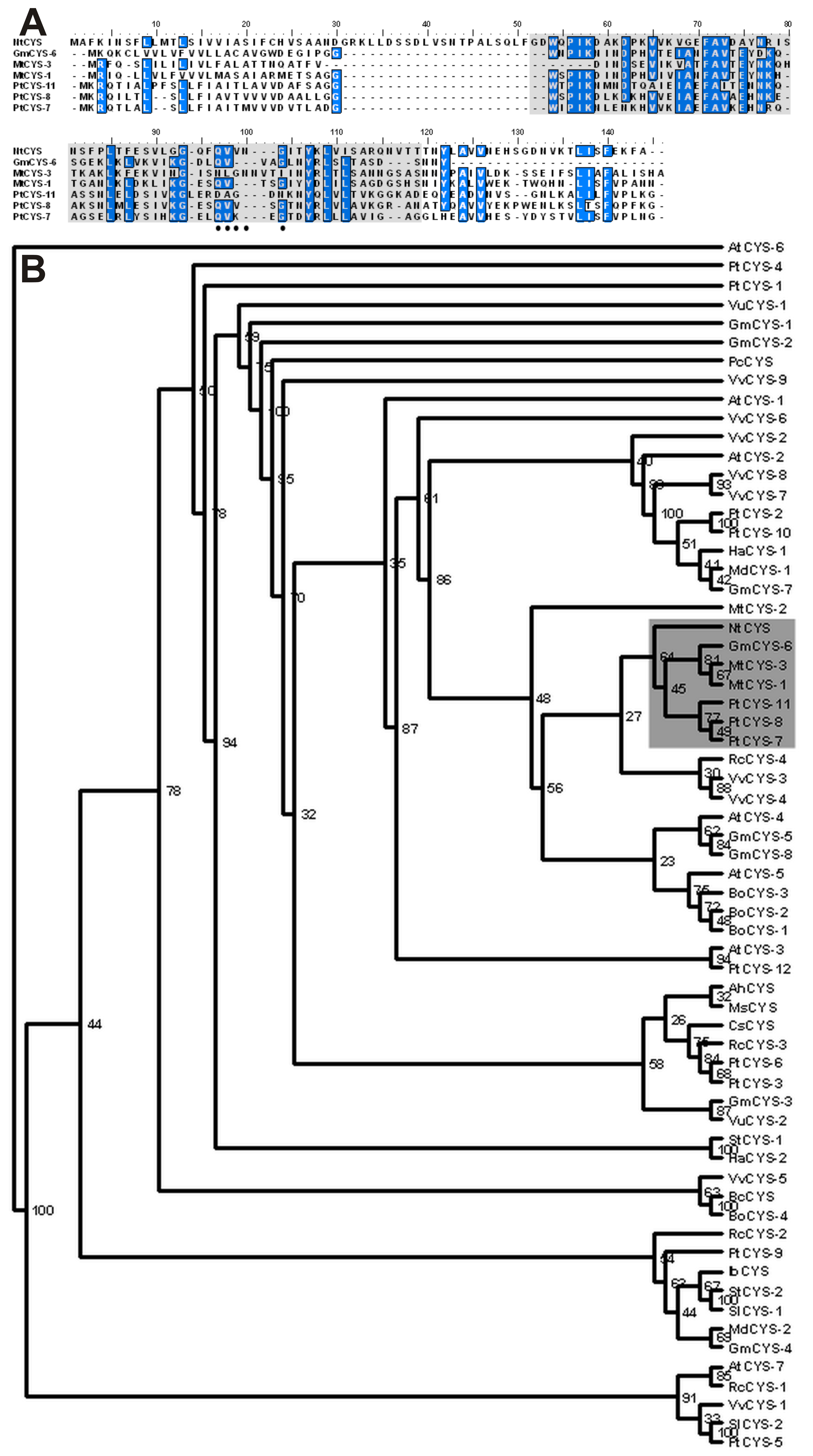

Supplement: Figure S1 — Sequence alignment and phylogenetic analysis of NtCYS protein. (A) Alignment of NtCYS with closely-related members of cystatin family, including G. max GmCYS-6 (accession number ACU14962), M. truncatula MtCYS-1 (ABD28732.1) and MtCYS-3 (ABD32914.1), and P. trichocarpa PtCYS-7 (XP_002308795), PtCYS-8 (XP_002307893), and PtCYS-11 (EEF07232). Identical amino acid residues are boxed. Cystatin-like domain is labelled with a shaded box. Black dots indicate QxVxG motif. (B) A bootstrap consensus of phylogenetic tree representing similarities of NtCYS protein sequence with those of Arachis hypogaea AhCYS (accession number: AAU21498), A. thaliana AtCYS-1 (AT5G12140), AtCYS-2 (AT2G31980), AtCYS-3 (AT3G12490), AtCYS-4 (AT4G16500), AtCYS-5 (AT5G47550), AtCYS-6 (At3g12490), AtCYS-7 (At5g05110), B. campestris BcCYS (S65071), B. oleracea BoCYS-1 (ABD64998), BoCYS-2 (ABD64972), BoCYS-3 (ABD64929), BoCYS-4 (AAL59842), Castanea sativa CsCYS (CAA11899), G. max GmCYS-1 (ACU14306), GmCYS-2 (CAI84599), GmCYS-3 (CAI84598), GmCYS-4 (BAA19610), GmCYS-5 (ACU19522), GmCYS-6 (ACU14962), GmCYS-7 (CAI84604), GmCYS-8 (CAI84601), Helianthus annuus HaCYS-1 (JE0308), HaCYS-2 (BAA95416), Ipomoea batatas IbCYS (AAD13812), Malus x domestica MdCYS-1 (AAO18638) and MdCYS-2 (AAO19652), M. truncatula MtCYS-1 (ABD28732.1), MtCYS-2 (ABD28593.2), MtCYS-3 (ABD32914.1), M. sativa MsCYS (AAZ98791.1), Pyrus communis PcCYS (AAB71505.1), P. trichocarpa PtCYS-1 (EEF09526), PtCYS-2 (EEE98761), PtCYS-3 (EEE82959), PtCYS-4 (XP_002336760), PtCYS-5 (XP_002319462), PtCYS-6 (XP_002314231), PtCYS-7 (XP_002308795), PtCYS-8 (XP_002307893), PtCYS-9 (XP_002303955), PtCYS-10 (XP_002301749), PtCYS-11 (EEF07232), PtCYS-12 (ABK94227), Ricinus communis RcCYS-1 (EEF36180), RcCYS-2 (XP_002525552), RcCYS-3 (XP_002523225), RcCYS-4 (EEF31214), Solanum lycopersicum SlCYS-1 (AAF23126), SlCYS-2 (ABG23376), SlCYS-3 (AAF23127), SlCYS-4 (ABY83981), SlCYS-5 (AAF23128), S. tuberosum StCYS-1 (AAA16120), StCYS-2 (ABA40456), Vigna unguiculata VuCYS [file pbio.1001655.s001.tif]

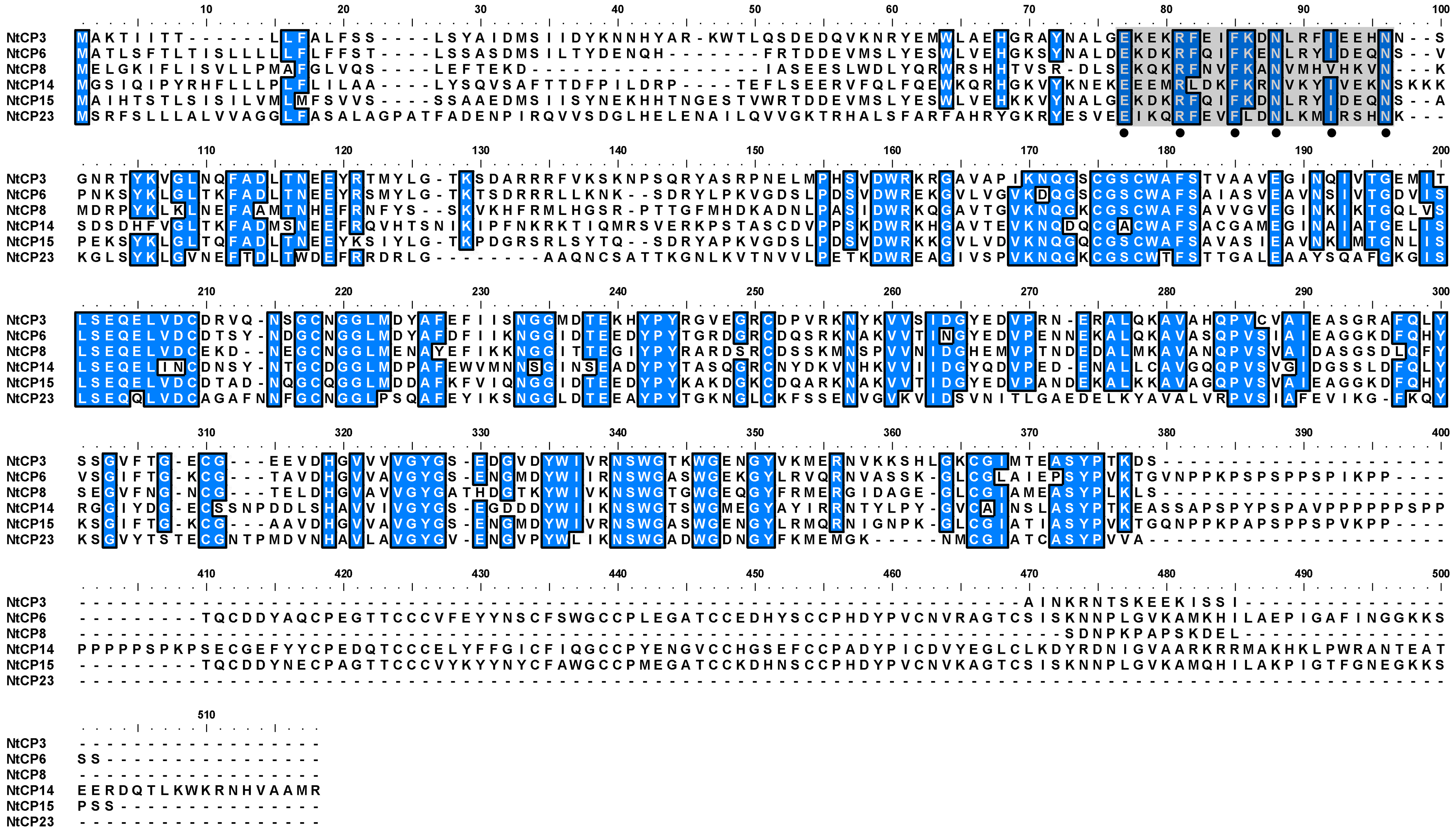

Supplement: Figure S2 — Sequence alignment of tobacco cathepsins. Identical amino acid residues are boxed. Black dots indicate non-contiguous ERFNIN motif. (TIFF) [file pbio.1001655.s002.tif]

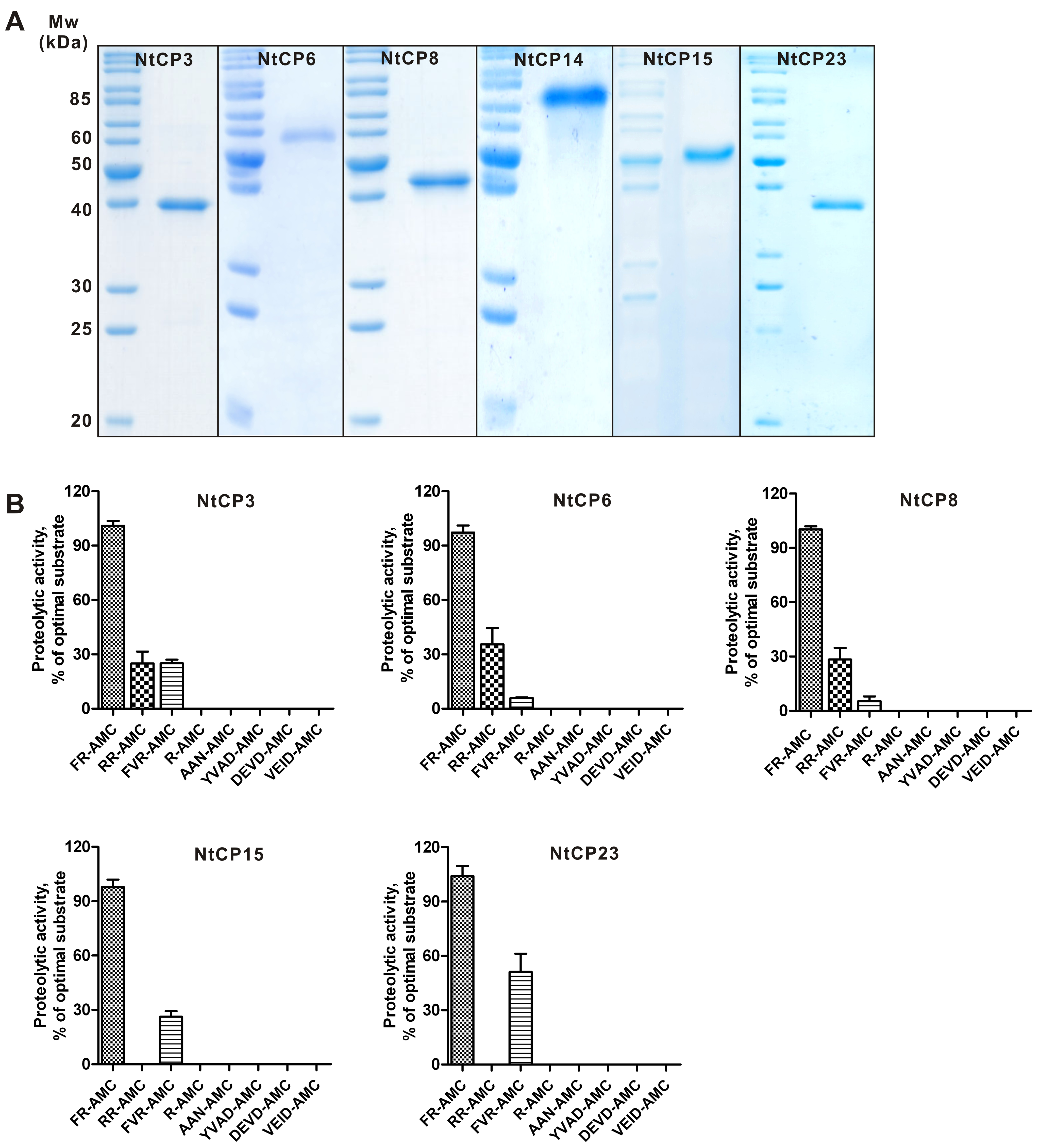

Supplement: Figure S3 — Purification and substrate specificity of tobacco cathepsins. (A) Coomassie blue stained SDS-PAGE gel showing purified cathepsins. (B) Proteolytic activities of recombinant cathepsins against different substrates. The activity of each cathepsin against different substrates is expressed as the percentage of its activity against substrate FR-AMC. Data represent the mean ± SE of three independent experiments. (TIF) [file pbio.1001655.s003.tif]

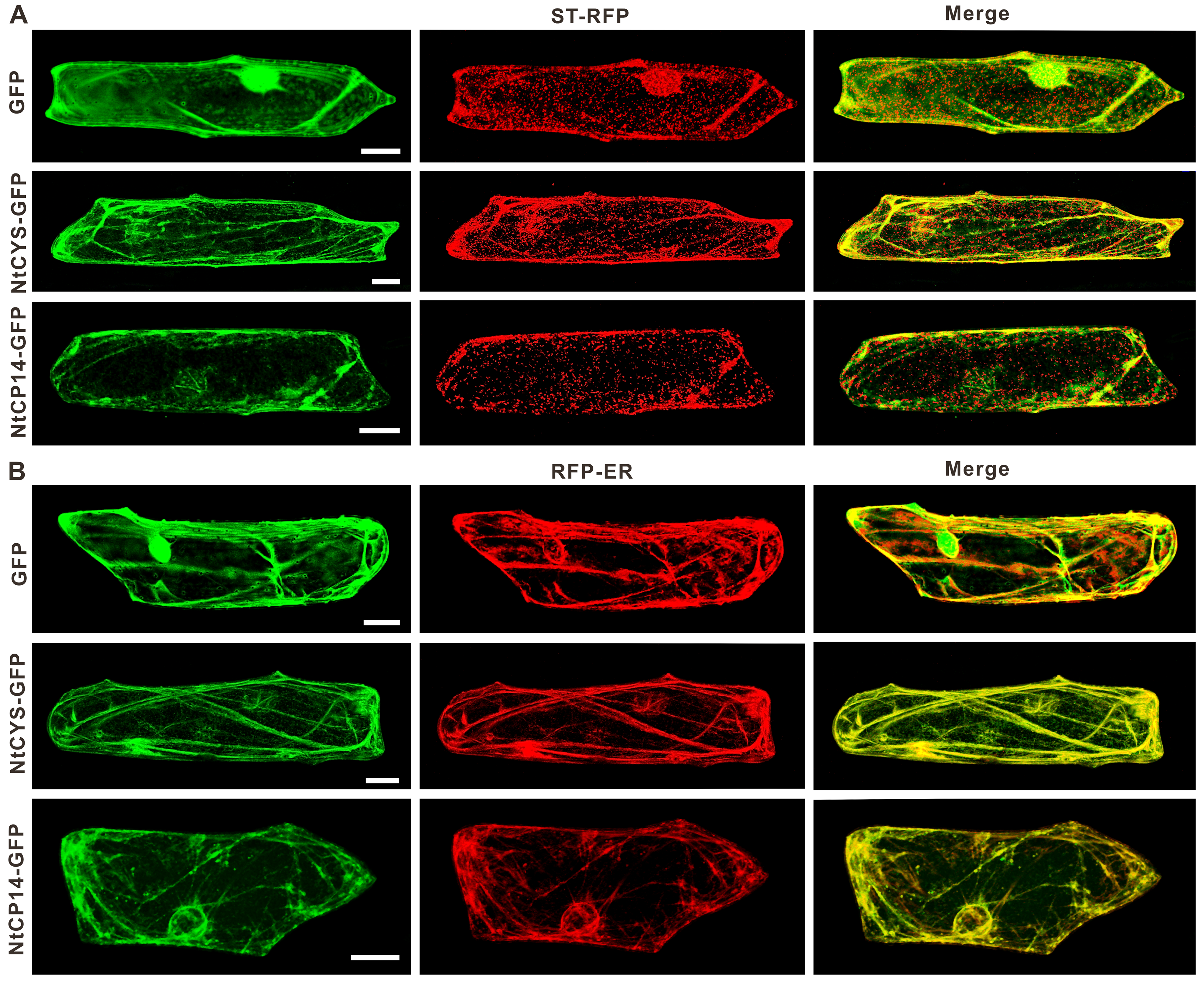

Supplement: Figure S4 — Co-localization analysis of NtCYS-GFP and NtCP14-GFP with Golgi and ER markers in onion epidermal cells. While no apparent co-localization was observed with Golgi marker ST-RFP (A), both proteins showed strong co-localization with ER marker RFP-ER (B). Scale bars, 50 µm. (TIF) [file pbio.1001655.s004.tif]

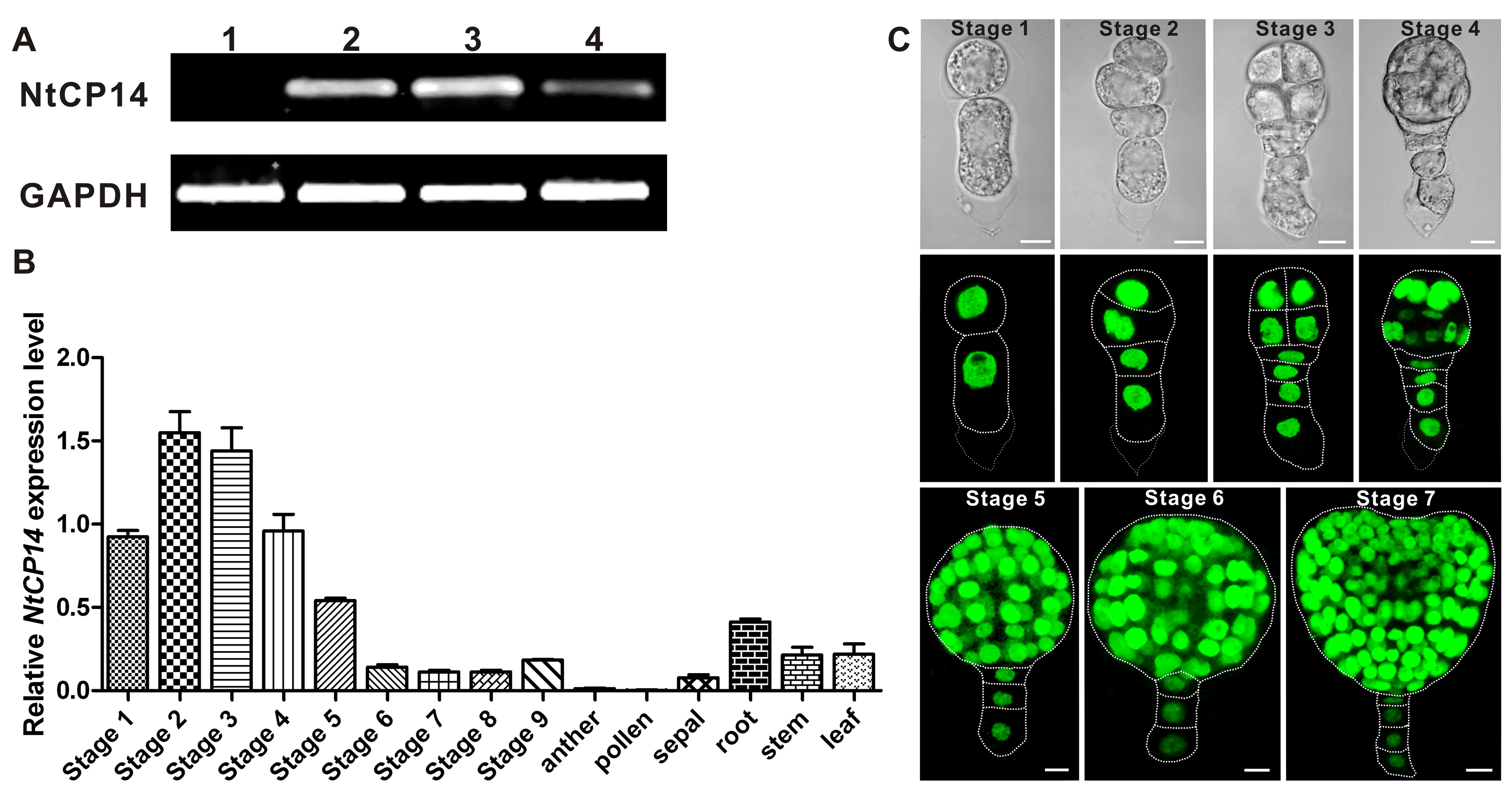

Supplement: Figure S5 — Expression pattern of NtCP14 . (A) Semi-quantitative RT-PCR analysis of NtCP14 in sperm cell (1), egg cell (2), zygote (3), and two-celled proembryo (4). Glyceraldehyde-3-phosphate dehydrogenase (GAPDH) was used as a control. (B) RT-qPCR analysis of NtCP14 in the embryos at stages 1 to 9, and in both floral and vegetative tissues. The expression level of NtCP14 in the embryos at stage 1 was set to 1. (C) Promoter activity of NtCP14 (proNtCP14::H2B-GFP expression) during embryogenesis. Scale bars, 10 µm. (TIF) [file pbio.1001655.s005.tif]

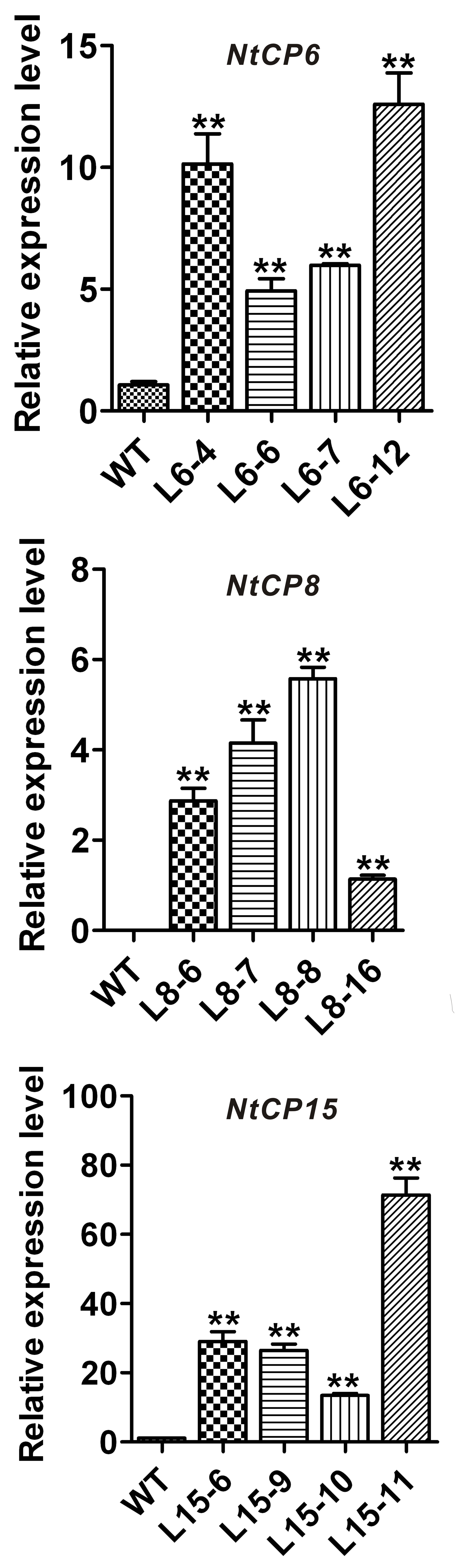

Supplement: Figure S6 — Overexpression of cathepsin L-like genes driven by promoter proZC1 . Enhanced expression of cathepsin L-like genes in transgenic lines, as measured by RT-qPCR. The expression level in the WT was set to l, except for NtCP8. Data represent the mean ± SE from three independent experiments. ** indicates statistical difference compared to WT (t-test, p<0.01). (TIF) [file pbio.1001655.s006.tif]

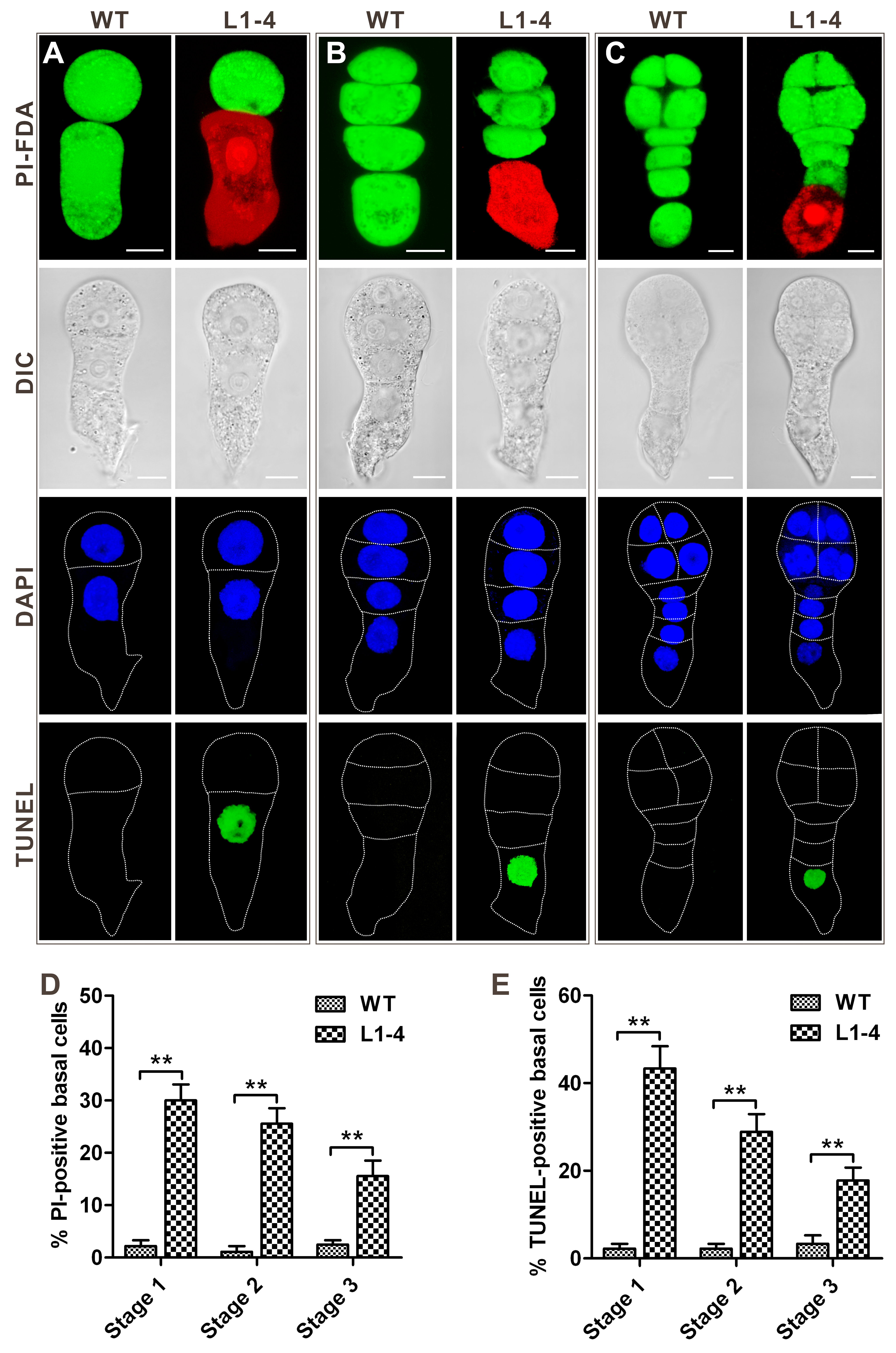

Supplement: Figure S7 — Overexpression of NtCP14 driven by proNtCYS induces precocious cell death in the basal cell lineage. (A–C) Cell viability and nuclear DNA fragmentation at developmental stages 1 (A), 2 (B), and 3 (C) from WT and NtCP14-overexpressing line L1-4. Scale bars, 10 µm. (D, E) The frequency of the proembryos (stages 1 and 2) and eight-celled embryos (stage 3) with PI-positive (D) and TUNEL-positive (E) basal cells in WT and NtCP14-overexpressing line L1-4. Data represent the mean ± SE from three independent experiments, with 30 proembryos or embryos per line analyzed in each experiment (n = 90). ** indicates statistical difference compared to WT (t-test, p<0.01). (TIF) [file pbio.1001655.s007.tif]

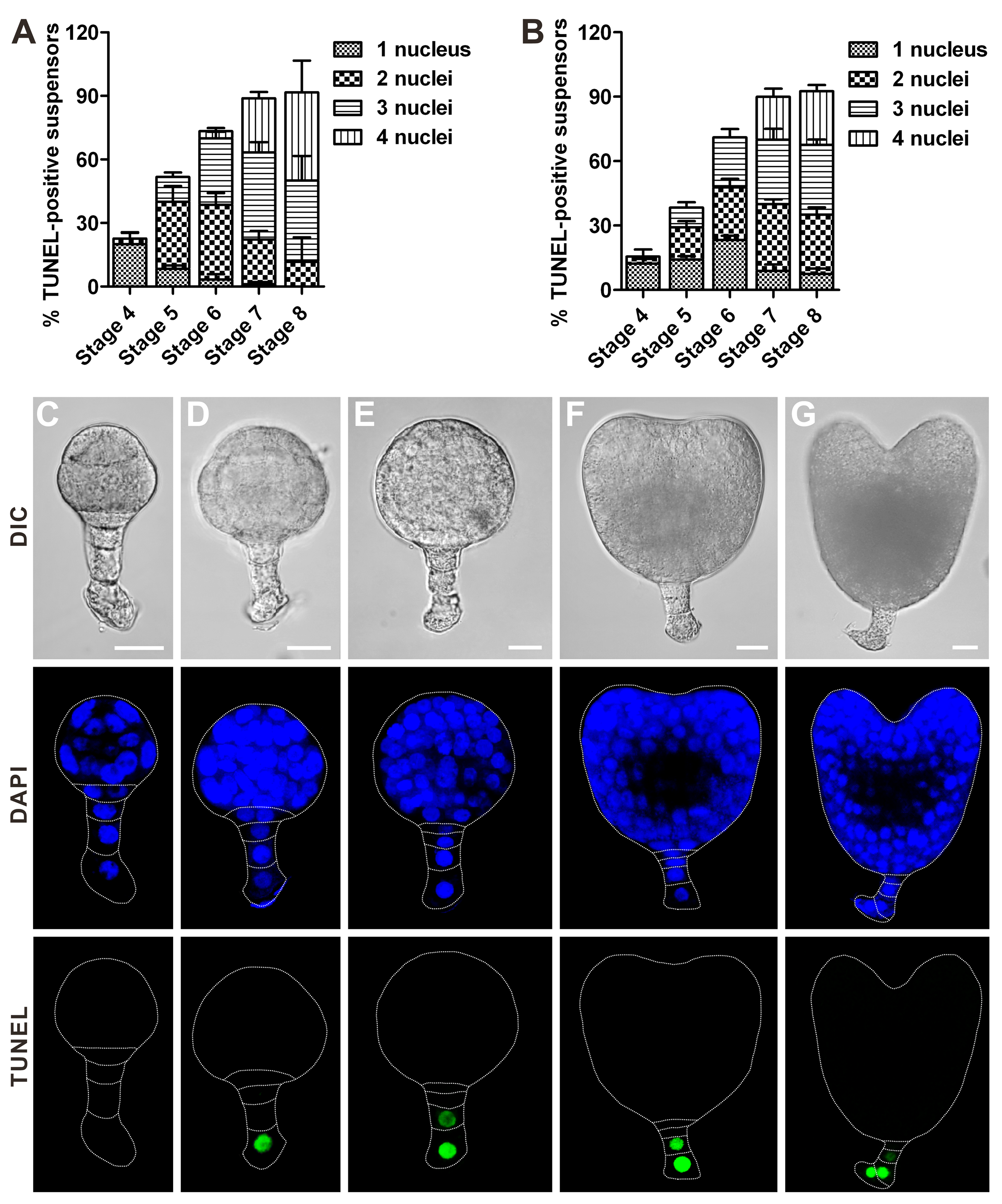

Supplement: Figure S8 — Upregulation of NtCYS or downregulation of NtCP14 delays the onset of suspensor PCD. (A) The frequency of suspensors containing indicated numbers of TUNEL-positive nuclei in NtCYS-overexpressing line L-2 at stages 4 to 8. Data represent the mean ± SE from three independent experiments, with 30 embryos per stage analyzed in each experiment (n = 90). (B) The frequency of suspensors containing indicated numbers of TUNEL-positive nuclei in NtCP14 RNAi line L3-15 at stages 4 to 8. Data represent the mean ± SE from three independent experiments, with 30 embryos per stage analyzed in each experiment (n = 90). (C–G) Representative examples of TUNEL-stained embryos in the NtCP14 RNAi lines at stages 4 to 8, respectively. Scale bars, 20 µm. (TIF) [file pbio.1001655.s008.tif]

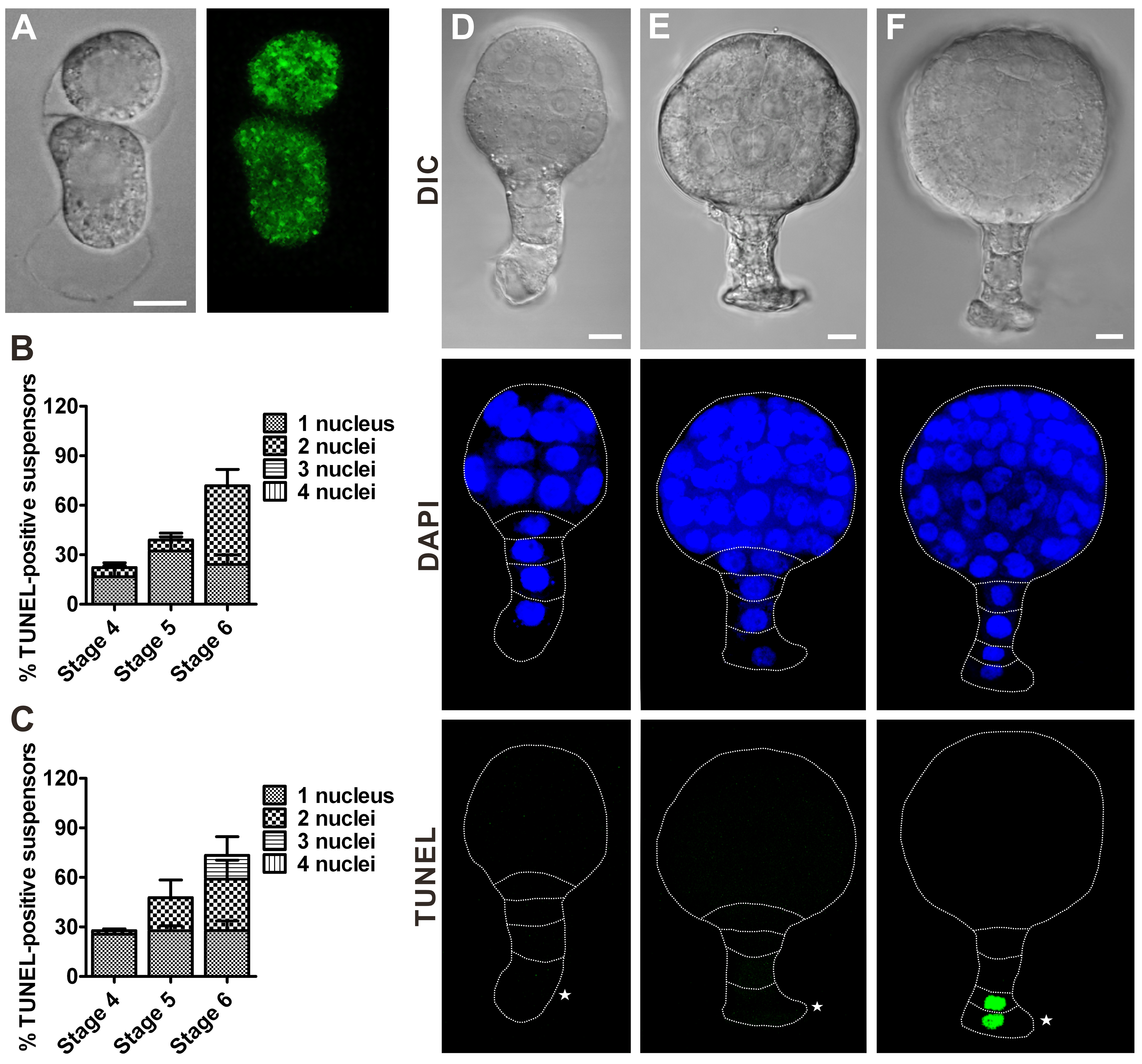

Supplement: Figure S9 — NtCYS-GFP expression delays the onset of suspensor PCD. (A) Expression of NtCYS-GFP driven by proZC1 in two-celled proembryos. Scale bars, 10 µm. (B) The frequency of suspensors containing indicated numbers of TUNEL-positive nuclei in NtCYS-GFP expressing line L-1 at stages 4 to 6. Data represent the mean ± SE from three independent experiments, with 30 embryos per stage analyzed in each experiment (n = 90). (C) The frequency of suspensors containing indicated numbers of TUNEL-positive nuclei in NtCYS-GFP expressing line L-2 at stages 4 to 6. Data represent the mean ± SE from three independent experiments, with 30 embryos per stage analyzed in each experiment (n = 90). (D–F) Representative examples of TUNEL-stained embryos in the NtCYS-GFP expressing lines at stages 4, 5, and 6, respectively. Scale bars, 10 µm. Asterisks indicate the basal cell. (TIF) [file pbio.1001655.s009.tif]

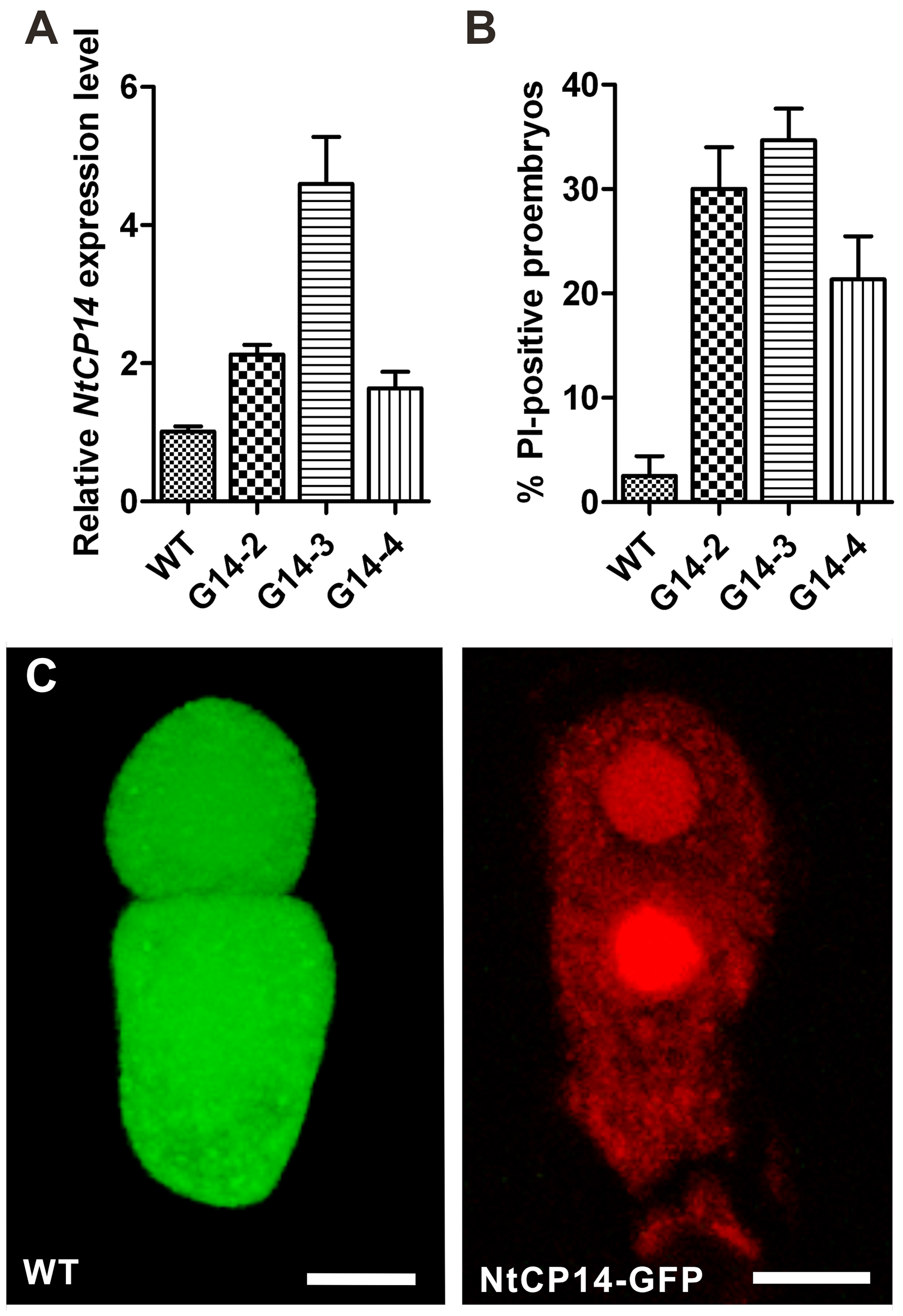

Supplement: Figure S10 — Expression of NtCP14-GFP induces precocious cell death. (A) The mRNA level of NtCP14 in pNtCP14::NtCP14-GFP transgenic lines. The expression level of NtCP14 in WT was set to l. (B) The frequencies of PI-positive two-celled proembryos in WT and NtCP14-GFP expression lines. Data represent the mean ± SE from three independent experiments with 50 proembryos per line analyzed in each experiment (n = 150). (C) PI-FDA stained two-celled proembryos from WT and pNtCP14::NtCP14-GFP line. Scale bars, 10 µm. (TIF) [file pbio.1001655.s010.tif]
