# Supplementary material for: A Bipartite Molecular Module Controls Cell Death Activation in the Basal Cell Lineage of Plant Embryos
Source: PLoS Biol. 2013 Sep 10;11(9):e1001655. doi: 10.1371/journal.pbio.1001655 (PMC3769231; doi:10.1371/journal.pbio.1001655)
Supplement: Text S1 — Additional details for methods. (DOC) [file pbio.1001655.s011.doc]

**Supporting Methods**

**Constructs and plant transformation**

For construction of a fusion between *NtCYS* and the GFP coding sequence (*ProNtCYS::NtCYS-GFP*), a 1.8-kb fragment containing the *NtCYS* promoter and the *NtCYS* coding sequence was amplified from genomic DNA and inserted into the pGEM-T Easy Vector (Promega). The fragment was cleaved with *Sac*I and *Xba*I and then ligated into the destination vector pART 27 for an eGFP fusion.

To generate *ProNtCP14::H2B-GFP* vector, a 1.9-kb *NtCP14* promoter fragment was amplified from genomic DNA and inserted into the pGEM-T Easy Vector (Promega). The fragment was cleaved with *Sac*I and *Xho*I and then ligated into the destination vector pART 27 upstream of H2B-GFP.

To generate different RNAi vectors, three fragments of *NtCYS* were amplified from genomic DNA and inserted between the *Xho*I and *Kpn*I sites (for the forward insert) or the *Xba*I and *Hin*dⅢ sites (for the reverse insert) of the pKANNIBAL vector. The first RNAi vector contained a 334-bp fragment from the 5’ UTR to 117 bp downstream of the ATG; the second RNAi vector contained a 420-bp coding region of *NtCYS*; and the third RNAi vector contained a 342-bp fragment from 118 bp downstream of the ATG to the end of the cDNA. To generate *NtCP14* RNAi vectors, three different fragments of *NtCP14* were amplified and inserted between the *Xho*I and *Kpn*I sites (for the forward insert) or the *Xba*I and *Hin*dⅢ sites (for the reverse insert) of the pKANNIBAL vector. The first RNAi vector contained a 556-bp fragment from ATG to 556 bp downstream of the ATG; the second RNAi vector contained a 517-bp fragment from 557 to 1073 bp downstream of the ATG; and the third RNAi vector contained a 563-bp fragment from 1074 bp downstream of the ATG to the end of the cDNA. The constructed vectors for *NtCYS* and *NtCP14* RNAi were digested using *Xho*I and *Not*I and cloned into the binary vector pART27 downstream of their native promoter, respectively.

To generate overexpression constructs for cysteine proteases, the respective full-length cDNAs were inserted into the pCAMBIA1302 vector with Lat52:GFP in the region of the *Hin*dⅢ and *Bst*EII sites downstream of the *ZC1* promoter isolated in our laboratory [1]. To generate overexpression construct for *NtCYS*, the full-length cDNA of *NtCYS* was inserted into the pCAMBIA1302 vector with Lat52:GFP in the region of the *Hin*dⅢ and *Bst*EII sites downstream of the 35S promoter.

*Agrobacterium tumefaciens* (strain LBA4404)-mediated transformation of *N. tabacum* plants was performed according to the method of Horsch et al. [2]. Regenerated plants were selected on Murashige and Skoog medium containing 250 mg l^−1^ carbenicillin and either 100 mg l^−1^ kanamycin or 50 mg l^−1^ hygromycin B.

#### Embryo isolation

To isolate tobacco embryos, seeds at different stages were collected and treated with 1% Cellulase R-10 and 0.8% Macerozyme R-10 (Yakult Honsha Co. Ltd, Tokyo, Japan) dissolved in 11% mannitol for 30 min at 25°C. Embryo sacs containing embryos at stage 1 to 3 were ﬁrstly released by gentle grinding with a small glass pestle. Early proembryos were then mechanically isolated from the embryo sacs after a second enzymatic treatment for 10 min with 0.25% Cellulase R-10 and 0.2% Macerozyme R-10 dissolved in 11% mannitol at 25°C. Embryos at stage 4 to 9 were directly isolated form seeds by gentle grinding with a small glass pestle after first enzymatic maceration as mentioned above. The microdissection was performed under an inverted microscope with a hand-made micropipette.

**Cell viability and DNA fragmentation analyses**

To evaluate cell viability and plasma membrane integrity, we stained embryos with fluorescein diacetate (FDA) and propidium iodide (PI). Isolated embryos were incubated in the solution containing 11% mannitol, 2 µg mL^-1^ FDA and 1 µg mL^-1^ PI for 15 min at room temperature and washed twice with 11% mannitol before observation. For detection of nuclear DNA fragmentation seeds were fixed in 4% paraformaldehyde and embryos were isolated using above described procedure. The embryos were incubated in a TUNEL reaction mixture prepared according to the manufacturer’s instructions (Promega).

***In situ* caspase-like activity assays**

To detect caspase-like activity in the embryos, Carboxy fluorescein FLICA Apoptosis Detection Kits (Immunochemistry Technologies, LLC) were used. Living embryos were isolated according to the above-described procedure and incubated in a reaction mixture diluted with 11% mannitol from 150×FLICA stock solution for 1 h at 30°C. After incubation with reaction mixture, the embryos were washed twice with washing buffer diluted with 11% mannitol from stock solution.

**Capillary electrophoresis for protease activity assay**

Capillaries were prepared as described [3]. The electrophoresis buffer contained of 15 mM sodium phosphate  (pH 6.0), and sample injection was performed in the hydrodynamic mode for 20 s with a sampling height of 9 cm. Sample separation was carried out at a constant voltage of 20 kV using a laboratory-built system based on an upright ﬂuorescence microscope (Olympus, Japan), a photomultiplier tube (PMT), a ±30 kV high-voltage DC power supply (Shanghai Institute of Nuclear Research, China), and an uncoated fused-silica capillary of 45 cm (34 cm to the detector window) × 50 µm i.d. × 365 µm o.d. (Yongnian Optical Conductive Fiber Plant, China).

**References**

1. Ning J, Peng XB, Qu LH, Xin HP, Yan TT, et al. (2006) Differential gene expression in egg cells and zygotes suggests that the transcriptome is restructed before the first zygotic division in tobacco. FEBS Lett 580: 1747-1752.

2. Horsch RB, Fry JE, Hoffmann NL, Eichholtz D, Rogers SG, et al. (1985) A simple and general method for transferring genes into plants. Science 227: 1229-1231.

3. Zhang LY, Sun MX (2004) Determination of histamine and histidine by capillary zone electrophoresis with pre-column naphthalene-2,3-dicarboxaldehyde derivatization and fluorescence detection. J Chromatogr A 1040: 133-140.
